# Chemical Composition, Anti-Breast Cancer Activity and Extraction Techniques of Ent-Abietane Diterpenoids from *Euphorbia fischeriana* Steud

**DOI:** 10.3390/molecules27134282

**Published:** 2022-07-03

**Authors:** Gang Chen, Tiancheng Ma, Yukun Ma, Cuicui Han, Jinling Zhang, Yu Sun

**Affiliations:** 1 Research Institute of Medicine and Pharmacy, Qiqihar Medical University, Bukui Road 333, Qiqihar 161006, China; cgsuperman.happy@163.com (G.C.); matiancheng2@126.com (T.M.); kuntengchongtian@163.com (Y.M.); zhangjinling0413@163.com (J.Z.); 2 School of Traditional Chinese Materia Medica, Shenyang Pharmaceutical University, Wenhua Road 103, Shenyang 110016, China; 3 College of Pharmacy, Qiqihar Medical University, Bukui Road 333, Qiqihar 161006, China

**Keywords:** ent-abietane diterpenoids, *Euphorbia fischeriana*, anti-breast cancer activity, response surface methodology, salting-out-assisted liquid–liquid extraction

## Abstract

Ent-abietane diterpenoids are the main active constituents of *Euphorbia fischeriana*. In the continuing search for new anti-breast cancer drugs, 11 ent-abietane diterpenoids (**1**–**11**) were isolated from *E. fischeriana*. The structures of these compounds were clearly elucidated on the basis of 1D and 2D NMR spectra as well as HRESIMS data. Among them, compound **1** was a novel compound, compound **10** was isolated from *Euphorbia* genus for the first time, compound **11** was firstly discovered from *E*. *fischeriana*. These compounds exhibited varying degrees of growth inhibition against the MCF-10A, MCF-7, ZR-75-1 and MDA-MB-231 cell lines in vitro. The experimental data obtained permit us to identify the roles of the epoxy group, hydroxyl group and acetoxyl group on their cytotoxic activities. Extraction is an important means for the isolation, identification, and application of valuable compounds from natural plants. To maximize yields of ent-abietane diterpenoids of *E. fischeriana*, 17-hydroxyjolkinolide B, jolkinolide B, 17-hydroxyjolkinolide A and jolkinolide A were selected as quality controls to optimize the salting-out-assisted liquid–liquid extraction (SALLE) by response surface methodology (RSM). The optimized conditions for SALLE were 0.47 g sodium dihydrogen phosphate, 5.5 mL acetonitrile and 4.5 mL water at pH 7.5. The experimental values of 17-hydroxyjolkinolide B, jolkinolide B, 17-hydroxyjolkinolide A and jolkinolide A (2.134, 0.529, 0.396, and 0.148 mg/g, respectively) were in agreement with the predicted values, thus demonstrating the appropriateness of the model.

## 1. Introduction

*Euphorbia fischeriana* Steud, belonging to the family of *Euphorbiaceae*, is a perennial herbaceous plant. Although the dried root of *E. fischeriana* is highly toxic, it has been used as traditional herbal medicine for thousands of years due to its therapeutic properties [1]. Numerous phytochemical studies have covered different types of compounds, including diterpenoids, triterpenoids, meroterpenoids, acetophenones, coumarins, steroids, phenolic acids and tannins [2,3,4,5,6,7]. Ent-abietane diterpenoids are the main bioactive constituents of *E. fischeriana*, which have been illustrated to have anti-tumor, antibacterial, anti-inflammatory and antiviral activities [8,9,10,11,12]. As part of our ongoing interest in discovering new anti-cancer agents from natural products, compounds isolated from *E. fischeriana* were of interest to us for their anti-breast cancer activity. In this study, 1 novel ent-abietane diterpenoid and 10 known ones were isolated. The anti-breast cancer activities of these compounds were tested and the structure–activity relationship was discussed.

Extraction is an important means of isolating, identifying, and applying valuable chemical compounds from natural plants. A number of extraction methods are available, including aqueous extraction, maceration extraction and solid-phase microextraction. Typically, these traditional extraction methods are very slow, costly, and inefficient. However, several new extraction methods have been discovered during recent years. Liquid–liquid extraction is a simple method with excellent extraction performance [13]. It extracts compounds according to their relative solubilities in aqueous phase and organic solvents. SALLE is an efficient extraction method using polar salt solvents, which shows broad analyte coverage, satisfactory reproducibility, acceptable recoveries, and low matrix interference [14]. Due to the wide range of prominent bioactivities and large number of ent-abietane diterpenoids in *E. fischeriana*, an efficient procedure needed to be developed. However, it remained challenging to optimize the extraction conditions. 17-Hydroxyjolkinolide B, jolkinolide B, 17-hydroxyjolkinolide A and jolkinolide A are abundant among the ent-abietane diterpenoids of *E. fischeriana* and attract increasing interest due to their profound biological activity. They were highly desirable for quality controls. RSM is a collection of statistical and mathematical techniques that have been successfully used to develop, improve, and optimize processes. RSM can be used to assess the influence of multiple factors and their interaction with one or more response variables. In this study, RSM employing Box–Behnken design (BBD) was applied to optimize the conditions for the extraction of ent-abietane diterpenoids by SALLE.

## 2. Results and Discussion

### 2.1. Structure Elucidation of Novel Compound

**Figure 1:**
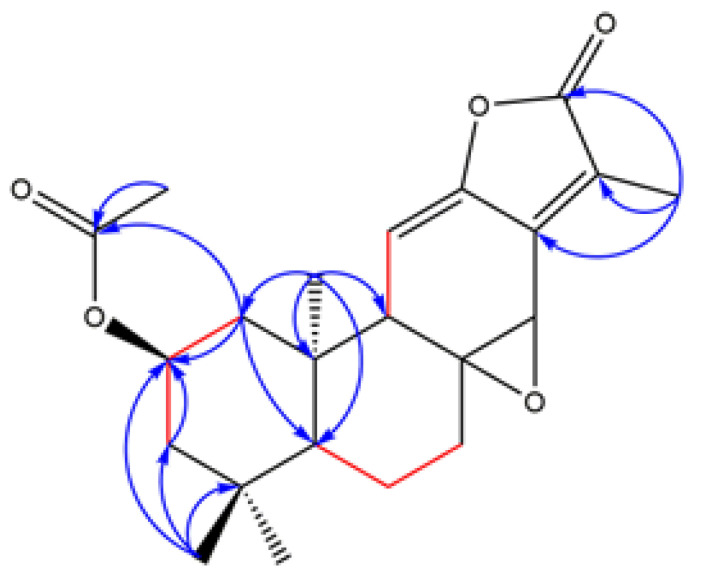
The key HMBC (H→C) and ^1^H–^1^H COSY (H—H) correlations of compound **1**.

**Figure 2:**
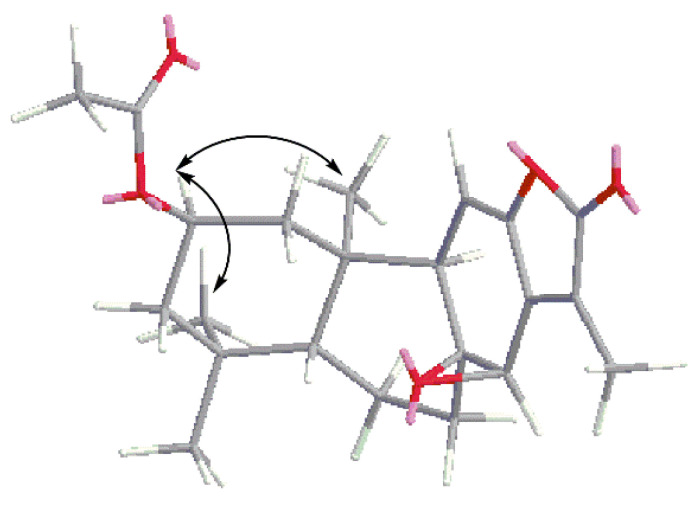
The key NOESY (H↔H) correlations of compound **1**.

Compound **1** was obtained as a white powder. The molecular formula of **1** was deduced to be C_22_H_28_O_5_ by HRESIMS analysis, displaying a protonated ion at *m*/*z* 373.2008 (calcd. [M + H]^+^, *m*/*z* 373.2010). The ^1^H NMR spectrum exhibited five methyl groups at *δ*_H_ 0.83 (3H, s), 0.96 (3H, s), 1.02 (3H, s) 2.04 (3H, s) and 2.07 (3H, s), one olefinic pronton 5.42 (1H, d, *J* = 5.34 Hz) and an oxygenated methine group 4.93 (1H, m). A combination of ^13^C NMR, DEPT and HSQC experiments indicated the presence of 22 carbon signals for compound **1**, confirming the presence of the above-mentioned groups and revealing other moieties, including an acetyl group (*δ*_C_ 21.6, 170.6) and an α,β-unsaturated-γ-lactone (*δ*_C_ 126.0, 144.8, 148.0, and 170.5). On the basis of spectroscopic data analysis (Table 1), compound **1** was deduced as being an ent-abietane diterpenoid, similar to jolkinolide A. Additionally, the ^13^C NMR data of **1** showed the signals for C-1, C-2, C-3, C-4 and C-10 shifted from *δ*_C_ 39.8, 18.4, 41.4, 33.4, 41.5 in jolkinolide A to *δ*_C_ 44.9, 68.0, 46.4, 35.1, 42.7 in **1**, which supposed that the acetoxy group was attached to ring A. The acetoxy group located at C-2 was speculated by the HMBC correlations from H-1 (*δ*_H_ 1.32), H-3 (*δ*_H_ 1.31) and Me-18 (*δ*_H_ 1.02) to C-2 (*δ*_C_ 68.0). It was further confirmed by the ^1^H–^1^H COSY correlations between H-1/H-2 and H-2/H-3. The key HMBC and ^1^H–^1^H COSY correlations were displayed in Figure 1. The correlations in the NOESY spectrum (Figure 2) between H-2/Me-20 and H-2/Me-19 suggested that H-2, Me-19 and Me-20 were α-orientation and Me-18 was β-orientation. On the basis of these data, the structure of compound **1** was identified as 2-acetoxyljolkinolide A.

### 2.2. Structure–Activity Relationship

**Figure 3:**
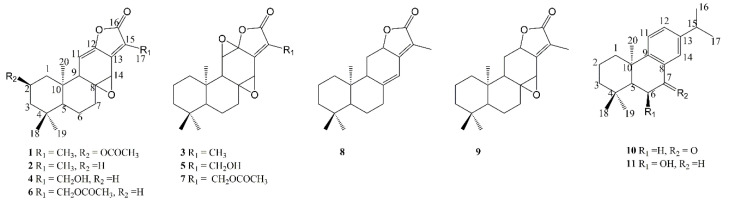
The isolated compounds (**1**–**11**).

The isolated diterpenoids were identified as 2-acetoxyljolkinolide A (**1**), jolkinolide A (**2**) [15], jolkinolide B (**3**) [16], 17-hydroxyljolkinolide A (**4**) [17], 17-hydroxyljolkinolide B (**5**) [17], 17-acetyljolkinolide A (**6**) [17], 17-acetyljolkinolide B (**7**) [18], jolkinolide E (**8**) [19], euphopilolide (**9**) [20], ent-abieta-8,11,13-triene-7-one (**10**) [21], 6β-hydroxy-ent-abieta-8,11,13-triene (**11**) [22] according to the literature. The spectra were shown in the Supplementary Materials. These structures were shown in Figure 3. Among them, compound **1** was a novel compound, compound **10** was isolated from *Euphorbia* genus for the first time, compound **11** was discovered from *E. fischeriana* for the first time. The cytotoxic activity of the isolated compounds (**1**–**11**) against MCF-10A, MCF-7, ZR-75-1 and MDA-MB-231 cell lines were displayed in Table 2. Paclitaxel was used as positive control. The structure–activity relationship was discussed.

In order to investigate the role of the epoxy group, the activities of compounds **2** and **3** were compared. The results indicated that the introduction of the epoxy group at C11-C12 considerably increased the cytotoxicity, as could also be seen from the activities of compounds **4** and **5**. However, comparison of the anti-breast cancer activities of compounds **8** and **9** on MCF-7, ZR-75-1 and MDA-MB-231 cell lines indicated that the epoxy group at C8-C14 had no significantly effect on the activity. The introduction of the C-17 hydroxyl group would increase the cytotoxicity which could be seen from the activities of compounds **2** and **4**. The conclusion was further validated by comparing the activities of compounds **3** and **5**. The activities of compounds **1** and **2** were investigated, which showed that the attachment of an acetoxy group at C-2 would increase the activity on MCF-10A, MCF-7 and MDA-MB-231 cell lines. To explore the effect of acetoxyl group at other sites, the activities of compounds **2**, **6**, **3** and **7**, which shared the same backbone but with an acetoxyl group added at C-17, respectively, were tested. The results showed that the C-17 acetoxyl group probably played a significant role in the cytotoxic activities, which could cause an increase in the activities. Then, we compared the activities of compounds **4** and **6**, and the results indicated that ent-abietane diterpenoid with acetoxyl group at C-17 were more activity than the ent-abietane diterpenoid with hydroxyl group at C-17 when there were no epoxy groups at C11-C12. Compounds **10** and **11** were specific types of ent-abietane diterpenoids without a D-ring. They showed moderate cytotoxic activity.

### 2.3. Method Validation

**Figure 4:**
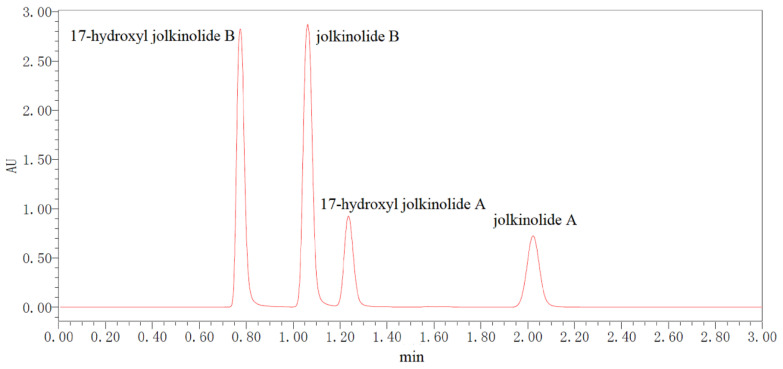
UPLC-UV chromatogram of the four ent-abietane diterpenoids.

The content determination of the four ent-abietane diterpenoids (Figure 4) was performed using UPLC-UV. The results of method validation were summarized in Table 3. The linearities of the standards were good for all analytes with the correlation coefficients more than 0.9997. The precision tests were carried out by injecting the same sample solution six times. Relative standard deviations (RSDs) were calculated to evaluate the precision. The results showed that the RSDs of intraday variations ranged from 0.26 to 0.45%. RSDs of interday variations ranged from 1.36 to 1.75%. RSDs of repeatability ranged from 2.00 to 4.65%. The recovery of the four diterpenoids were ranged from 95.1 to 104.9%. The results above indicated that the analytical method was suitable for the determination of the four ent-abietane diterpenoids of *E. fischeriana*.

### 2.4. Single-Factor Experiments

#### 2.4.1. Type of Salt and Solvent

In the process of SALLE, 32 different kinds of salts including ferrous sulfate, ferric trichloride, magnesium chloride, ammonia chloride, sodium carbonate anhydrous, disodium hydrogen phosphate, ammonium sulfate, sodium hydroxide, aluminium nitrate, sodium chloride, sodium nitrite, sodium oxalate, potassium sulphate, ammonium acetate, trisodium citrate, citric acid, ammonia sulfamate, oxalic acid dihydrate, potassium nitrate, ammonium formate dihydrate, potassium peroxodisulfate, sodium lauryl sulfate, potassium chloride, zinc sulfate, sodium hydrogen sulfate hydrate, sodium 1-decanesulfonate, ethylene diamine tetraacetic acid, calcium chloride, ammonium dihydrogen phosphate, anhydrous sodium sulfite, tetrabutylammonium bisulfate and sodium bicarbonate were used to screen the extraction condition. What is more, it is important to choose proper solvent in the extraction procedure, which would have big effect on the extraction ratio. To select the most appropriate one, methanol, ethanol and acetonitrile were evaluated based on the extraction efficiency of the target analytes. Considering the formation of aqueous two-phase and extraction ratio of ent-abietane diterpenoids, sodium dihydrogen phosphate and acetonitrile were chosen to further optimize the extraction conditions.

#### 2.4.2. Dosage of Salt

**Figure 5:**
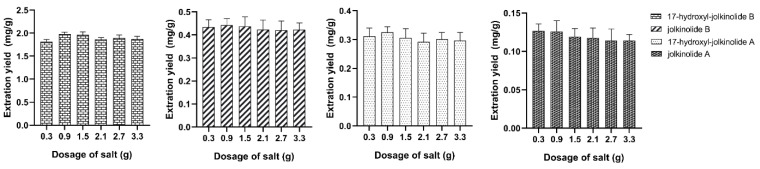
The effect of dosage of salt.

To look deep into the method optimization of SALLE, the influence of dosage of salt on extraction performance was investigated. The dosage of sodium dihydrogen phosphate at 0.3, 0.9, 1.5, 2.1, 2.7 and 3.3 g were tested. We found that the homogeneous solution did not tend to separate into two layers when the dosage of sodium dihydrogen phosphate less than 0.3 g. It showed that high dosage of salt addition favored phase separation. However, the proportion of the two layers remained essentially unchanged when the dosage of sodium dihydrogen phosphate was over 2.1 g. As the results of extraction ratio shown in Figure 5, the extraction ratio of the four target analytes were increased with increasing dosage of salt from 0.3 to 0.9 g. The extraction efficiency decreased when dosage of salt was over 0.9 g. Therefore, dosages of salt ranging from 0.3 to 1.5 g were chosen for the subsequent extraction procedure.

#### 2.4.3. Time of Vortex

**Figure 6:**
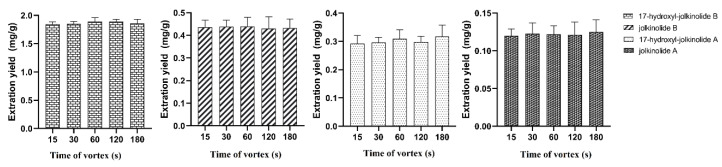
Time of vortex.

To evaluate the performance of vortex and ultrasound in the process of SALLE, the extraction ratio of the four target analytes were discussed by these two methods. It was observed that the vortex method had higher extraction efficiency in contrast with ultrasonic method. Then, the time of vortex was further investigated. Five time intervals at 15, 30, 60, 120 and 180 s were tested to evaluate the effect of vortex time. Figure 6 indicated that the vortex time had little influence on the extraction ratio.

#### 2.4.4. Weights of Samples

**Figure 7:**
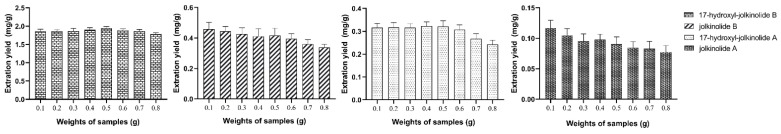
The effect of weights of samples.

Different weights of samples (0.1–0.8 g) were selected to investigate the effect of sample weight. As the results shown in Figure 7, the extraction ratio of the four target analytes decreased when the weight of sample was over 0.5 g.

#### 2.4.5. Acetonitrile to Water Ratio

**Figure 8:**
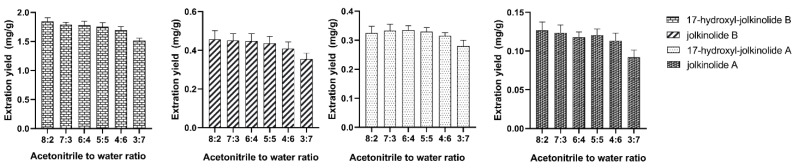
The effect of acetonitrile-to-water ratio.

In order to optimize the extraction conditions, the effect of acetonitrile-to-water ratio was studied. It did not tend to separate into two layers when acetonitrile-to-water ratio less than 3:7 or more than 8:2. Thus, acetonitrile to water ratios at 8:2, 7:3, 6:4, 5:5, 4:6 and 3:7 were tested to evaluate the effect of liquid to liquid ratio. As the results shown in Figure 8, the acetonitrile to water ratio at 8:2, 7:3 and 6:4 was chosen to further optimize the extraction conditions.

#### 2.4.6. pH of Water

**Figure 9:**
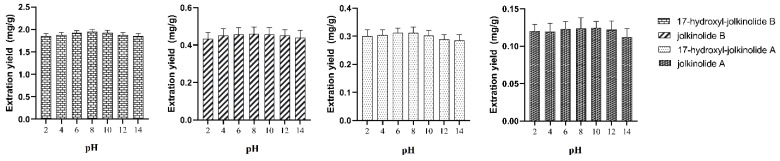
The effect of pH.

To select the most appropriate pH of water, pH valued at 2, 4, 6, 8, 10, 12 and 14 were evaluated based on the extraction efficiency of the target analytes. As the results show in Figure 9, it had higher extraction ratio when the pH of water ranged from 6 to 10.

### 2.5. Optimization of the Extraction Conditions Using BBD

#### 2.5.1. Response Surface Methodology

In order to optimize the extraction conditions, BBD combined with RSM was utilized. The operating conditions and experiment data were shown in Table 4. These data were analyzed using Design Expert 8.0.6 software for second-order polynomial regression analysis and ANOVA (Table 5). This mathematical regression models were shown below in terms of coded where *X*_1_, *X*_2_ and *X*_3_ were dosage of salt, pH of water, and acetonitrile to water ratio. *Y*_1_, *Y*_2_, *Y*_3_ and *Y*_4_ were the contents of 17-hydroxyjolkinolide B, jolkinolide B, 17-hydroxyjolkinolide A and jolkinolide A. The relationship between the contents of 17-hydroxyjolkinolide B (*R*^2^ = 0.9080), jolkinolide B (*R*^2^ = 0.9674), 17-hydroxyjolkinolide A (*R*^2^ = 0.9298) and jolkinolide A (*R*^2^ = 0.9579) and the extraction parameters (coded factors) are given below:

*Y*_1_ = 1.98 − 0.013*X*_1_ + 7.661 × 10^−3^*X*_2_ − 0.015*X*_3_ + 0.029*X*_1_*X*_2_ + 0.052*X*_1_*X*_3_ + 0.022*X*_2_*X*_3_ + 0.033*X*_1_^2^ − 0.020*X*_2_^2^ + 0.018*X*_3_^2^

*Y*_2_ = 0.46 − 1.065 × 10^−3^*X*_1_ + 2.274 × 10^−3^*X*_2_ − 0.016*X*_3_ + 4.797 × 10^−3^*X*_1_*X*_2_ + 0.019*X*_1_*X*_3_ + 6.949 × 10^−3^*X*_2_*X*_3_ + 7.299 × 10^−3^*X*_1_^2^ − 8.932 × 10^−3^*X*_2_^2^ + 7.907 × 10^−3^*X*_3_^2^

*Y*_3_ = 0.34 + 4.035 × 10^−3^*X*_1_ + 0.010*X*_2_ − 0.023*X*_3_ + 8.405 × 10^−3^*X*_1_*X*_2_ + 0.011*X*_1_*X*_3_ + 0.013 *X*_2_*X*_3_ − 1.234 × 10^−3^*X*_1_^2^ − 0.010*X*_2_^2^
+ 3.210 × 10^−3^*X*_3_^2^

*Y*_4_ = 0.13 − 2.270 × 10^−4^*X*_1_ − 2.950 × 10^−4^*X*_2_ − 7.418 × 10^−3^*X*_3_ − 4.925 × 10^−4^*X*_1_*X*_2_ + 6.712 × 10^−3^*X*_1_*X*_3_ + 2.547 × 10^−3^*X*_2_*X*_3_ + 2.378 × 10^−3^*X*_1_^2^ − 1.913 × 10^−3^*X*_2_^2^ + 6.303 × 10^−4^*X*_3_^2^

**Figure 10:**
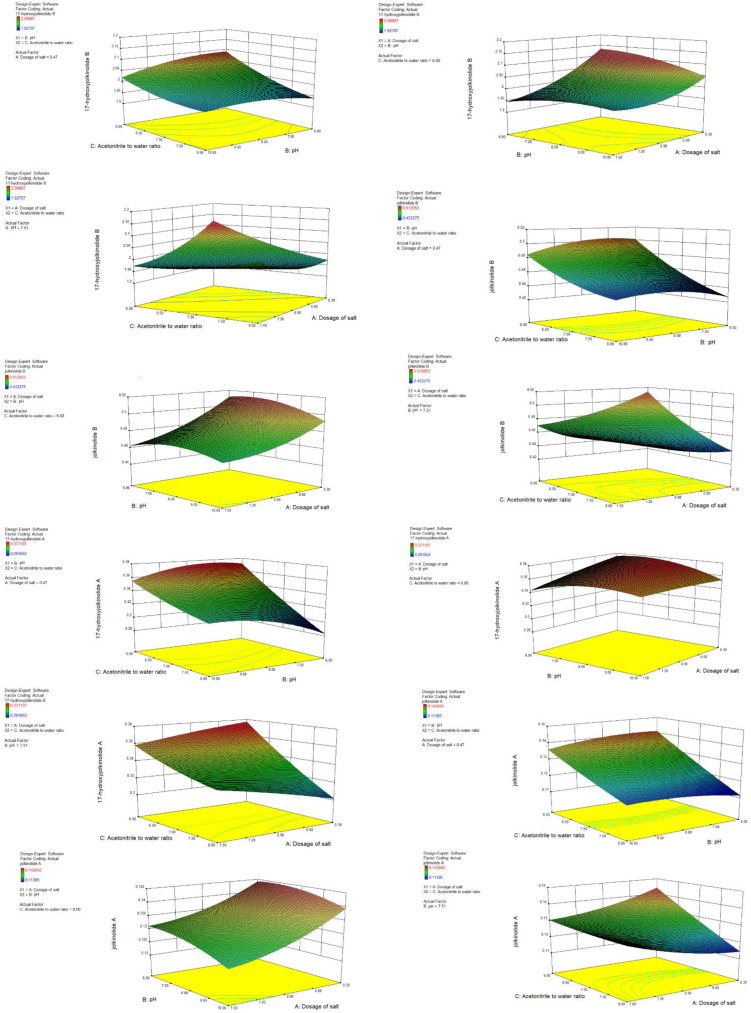
3D plots of the response surface for the contents of four diterpenoids as related to dosage of salt, pH of water, and acetonitrile to water ratio.

The *p*-values of the four quadratic models were all significant (*p* < 0.01). Lack of fits were not significant. It indicates that the model was applicable to describe the responses of the experiment. Figure 10 showed 3D plots of the response surface for the contents of four diterpenoids as related to dosage of salt, pH of water, and acetonitrile-to-water ratio. The optimized condition for extraction was 0.9 g sodium dihydrogen phosphate, 5.5 mL acetonitrile and 4.5 mL water with pH 7.5. Under these parameters, the predicted extraction contents for 17-hydroxyjolkinolide B, jolkinolide B, 17-hydroxyjolkinolide A and jolkinolide A were 2.134, 0.529, 0.396 and 0.148 mg/g, respectively.

#### 2.5.2. Verification of the Models

In order to verify the suitability of the predicted response values, verification experiments were performed under the optimized conditions in three replicates. The extraction contents for jolkinolide A, jolkinolide B, 17-hydroxyjolkinolide A and 17-hydroxyjolkinolide B were 2.217, 0.501, 0.374, and 0.151 mg/g, which were very close to the predicted value. This indicated that the established quadratic models were statistically reliable and reasonable.

### 2.6. Comparison with Conventional Ultrasonic-Assisted Extraction

To evaluate the performance of the proposed SALLE, ultrasonic-assisted extraction was introduced to compare the extraction. The extraction contents for jolkinolide A, jolkinolide B, 17-hydroxyjolkinolide A and 17-hydroxyjolkinolide B were 1.934, 0.489, 0.352 and 0.147 mg/g. It was obviously observed that the developed method had higher amount in contrast with ultrasonic-assisted extraction. Overall, the results indicated that the SALLE was a rapid and effective method for the extraction.

In conclusion, SALLE is an efficient, timesaving and energy-saving extract method. It does not need to be heated to high temperatures, which is suitable for thermolabile substances. Extraction of ent-abietane diterpenoids from *E. fischeriana* using SALLE is reported for the first time. Whether this method is suitable for other kind of compounds from *E. fischeriana* needs to be discussed in the further study.

## 3. Materials and Methods

### 3.1. Plant Material

The herbs of *E. fischeriana* were bought from Xianhe Pharmaceutical Company and verified as genuine ones by Professor Lina Guo (Voucher number: 20200503). A voucher specimen has been deposited at the Research Institute of Medicine and Pharmacy of Qiqihar Medical University.

### 3.2. Apparatus and Reagents

HRESIMS were determined by Q-TOF-MS system (SCIEX, Framingham, MA, USA). The NMR spectra were recorded on Bruker Ascend 600 NMR spectrometer (Bruker, Bremen, Germany), using TMS as an internal standard. HPLC purifications were performed on an analytical reversed-phase column (YMC-packed C_18_, 250 mm × 10 mm, 5 μm) (YMC, Tokyo, Japan) using a Waters 2535 Pump and detected with a Waters 2414 refractive index detector. Column chromatography was performed with silica gel (100–200, 200–300 mesh, Qingdao Haiyang Chemical Co., Ltd., Qingdao, China), ODS-A gel (S-20 μm, Beijing Jinouya Chemical Co., Ltd., Beijing, China), TLC was carried out on silica gel GF254 (Qingdao Haiyang Chemical Co., Ltd., Qingdao, China). Methanol was purchased from Merck Company. Ethanol, petroleum ether, ethyl acetate and dichloromethane were purchased from Tianjin Fuyu chemical Co. Ltd. (Tianjin, China)

### 3.3. Extraction and Isolation

The air-dried roots of *E. fischeriana* (30 kg) were extracted with 84 L EtOH by cold-dipping method (5 times, 24 h each time). The EtOH extract was evaporated under reduced pressure to provide a residue that was suspended in water and then extracted with petroleum ether, ethyl acetate, and n-butanol successively. The ethyl acetate fraction was subjected to CC (silica gel; petroleum ether/ethyl acetate 0:100→100:0) to afford five fractions (Frs.A–E). Fr.B was subjected to CC (reversed-phase C_18_ silica gel; MeOH/H_2_O 50:50→100:0) and afforded seven fractions (Frs.B.1–B.7). Fr.B.3 was further purified by RP-HPLC with MeOH/H_2_O as mobile phase (80:20) to afford **2** (124.5 mg), **4** (43.8 mg). Fr.B.4 was subjected to CC (silica gel; petroleum ether/ethyl acetate 0:100→100:0) to afford five fractions (Frs.B.4.1–B.4.8). Fr.B.6 was further purified by RP-HPLC with MeOH/H_2_O as mobile phase (85:15) to afford **8** (13.1 mg), **9** (8.7 mg). Fr.C was subjected to CC (reversed-phase C_18_ silica gel; MeOH/H_2_O 50:50→100:0) and afforded 11 fractions (Frs. C.1–C.11). Fr.C.3 was crystallized to afford **3** (521.9 mg). Fr.C.5 was subjected to CC (silica gel; petroleum ether/ethyl acetate 0:100→100:0) and afforded nine fractions (Frs.C.5.1–C.5.9). Fr.C.5.2 was crystallized to afford **5** (439.8 mg). Fr.C.7 was subjected to CC (silica gel; petroleum ether/ethyl acetate 0:100→100:0) and afforded nine fractions (Frs.C.7.1–C.7.9). Fr.C.7.3 was further purified by RP-HPLC with MeOH/H_2_O as mobile phase (75:25) to afford **1** (4.6 mg). Fr.C.7.5 was further purified by RP-HPLC with MeOH/H_2_O as mobile phase (70:30) to afford **6** (6.7 mg) and **7** (5.2 mg). Fr.C.7.7 was further purified by RP-HPLC with MeOH/H_2_O as mobile phase (70:30) to afford **10** (11.5 mg) and **11** (8.8 mg).

2-Acetoxyljolkinolide A (**1**): white amorphous powder, HRESIMS *m*/*z* 373.2008 [M + H]^+^ (calcd. for C_22_H_29_O_5_, 373.2010); ^1^H and ^13^C NMR data, see Table 1.

Jolkinolide A (**2**): white amorphous powder, HRESIMS *m*/*z* 315.1956 [M + H]^+^ (calcd. for C_20_H_27_O_3_, 315.1955); ^1^H NMR (600 MHz, CDCl_3_): *δ*_H_ 0.70 (3H, s H-20), 0.83 (3H, s, H-19), 0.92 (3H, s H-18), 2.03 (3H, s, H-17), 2.61 (1H, d, *J* = 5.3 Hz, H-9), 3.70 (1H, s, H-14), 5.43 (1H, d, *J* = 5.4 Hz, H-11); ^13^C NMR (150 MHz, CDCl_3_): *δ*_C_ 41.6 (C-1), 18.6 (C-2), 40.0 (C-3), 33.6 (C-4), 53.6 (C-5), 21.0 (C-6), 34.3 (C-7), 61.3 (C-8), 51.9 (C-9), 41.6 (C-10), 104.2 (C-11), 147.6 (C-12), 145.2 (C-13), 54.6 (C-14), 125.3 (C-15), 170.7 (C-16), 8.8 (C-17), 33.6 (C-18), 22.1 (C-19), 15.1 (C-20).

Jolkinolide B (**3**): white amorphous powder, HRESIMS *m*/*z* 331.1903 [M + H]^+^ (calcd. for C_20_H_27_O_4_, 331.1904); ^1^H NMR (600 MHz, CDCl_3_): *δ*_H_ 0.80 (3H, s, H-20), 0.83 (3H, s, H-19), 0.91 (3H, s, H-18), 2.06 (3H, s, H-17), 2.26 (1H, s, H-9), 3.67 (1H, s, H-14), 4.01 (1H, s, H-11); ^13^C NMR (150 MHz, CDCl_3_): *δ*_C_ 41.4 (C-1), 18.5 (C-2), 39.2 (C-3), 33.7 (C-4), 53.6 (C-5), 21.0 (C-6), 35.7 (C-7), 66.2 (C-8), 48.1 (C-9), 39.3 (C-10), 61.1 (C-11), 85.3 (C-12), 148.8 (C-13), 55.4 (C-14), 130.4 (C-15), 169.7 (C-16), 8.9 (C-17), 33.6 (C-18), 22.0 (C-19), 15.5 (C-20).

17-Hydroxyljolkinolide A (**4**): white amorphous powder, HRESIMS *m*/*z* 331.1908 [M + H]^+^ (calcd. for C_20_H_27_O_4_, 331.1904); ^1^H NMR (600 MHz, CDCl_3_): *δ*_H_ 0.73 (3H, s, H-20), 0.85 (3H, s, H-19), 0.94 (3H, s, H-18), 2.64 (1H, d, *J* = 5.3 Hz, H-9), 4.04 (1H, d, *J*=5.4 Hz, H-14), 4.64 (2H, s, H-17), 5.57 (1H, d, *J* = 5.4 Hz, H-11); ^13^C NMR (150 MHz, CDCl_3_): *δ*_C_ 41.7 (C-1), 18.6 (C-2), 40.1 (C-3), 33.7 (C-4), 53.6 (C-5), 21.0 (C-6), 34.2 (C-7), 61.5 (C-8), 52.0 (C-9), 41.7 (C-10), 106.7 (C-11), 147.5 (C-12), 146.8 (C-13), 54.6 (C-14), 127.7 (C-15), 169.4 (C-16), 56.5 (C-17), 33.7 (C-18), 22.1 (C-19), 15.3 (C-20).

17-Hydroxyljolkinolide B (**5**): white amorphous powder, HRESIMS *m*/*z* 347.1848 [M + H]^+^ (calcd. for C_20_H_27_O_5_, 347.1853); ^1^H NMR (600 MHz, CDCl_3_): *δ*_H_ 0.85 (3H, s, H-20), 0.85 (3H, s, H-19), 0.93 (3H, s, H-18), 2.29 (1H, s, H-9), 4.06 (1H, br s, H-11),4.11 (1H, s, H-14), 4.65 (2H, d, *J* = 2.9 Hz, H-17); ^13^C NMR (150 MHz, CDCl_3_): *δ*_C_ 41.5 (C-1), 18.6 (C-2), 39.3 (C-3), 33.7 (C-4), 53.7 (C-5), 21.0 (C-6), 33.7 (C-7), 67.0 (C-8), 48.0 (C-9), 39.4 (C-10), 61.7 (C-11), 85.6 (C-12), 151.2 (C-13), 55.4 (C-14), 133.2 (C-15), 168.3 (C-16), 56.7 (C-17), 33.7 (C-18), 22.1 (C-19), 15.7 (C-20).

17-Acetyljolkinolide A (**6**): yellowish oil, HR-ESI-MS *m*/*z* 373.2009 [M + H]^+^ (calcd. for C_22_H_29_O_5_, 373.2010); ^1^H NMR (600 MHz, CDCl_3_): *δ*_H_ 0.72 (3H, s, Me-18), 0.87 (3H, s, Me-17), 0.92 (3H, s, Me-19), 2.11 (3H, s, Me-2’), 3.96 (1H, s, H-14), 4.99 (2H, AB q, *J* = 13.6 Hz, H-17), 5.62 (1H, d, *J* = 5.4 Hz, H-11); ^13^C NMR (150 MHz, CDCl_3_): *δ*_C_ 40.2 (C-1), 18.7 (C-2), 41.7 (C-3), 33.9 (C-4), 53.8 (C-5), 20.1 (C-6), 34.1 (C-7), 61.5 (C-8), 52.2 (C-9), 41.8 (C-10), 107.8 (C-11), 149.7 (C-12), 147.4 (C-13), 54.5 (C-14), 122.9 (C-15), 170.7 (C-16), 55.7 (C-17), 33.7 (C-18), 22.1 (C-19), 15.3 (C-20), 20.0 (C-1’), 168.8 (C-2’).

17-Acetyljolkinolide B (**7**): yellowish oil, HRESIMS *m*/*z* 389.1958 [M + H]^+^ (calcd. for C_22_H_29_O_6_, 389.1959); ^1^H NMR (600 MHz, CDCl_3_): *δ*_H_ 0.79 (3H, s, Me-20), 0.86 (3H, s, Me-19), 0.94 (3H, s, Me-18), 2.11 (3H, s, Me-2’), 3.99 (1H, s, H-14), 4.06 (1H, d, *J* = 0.84 Hz, H-11), 4.99 (2H, d, *J* = 13.6 Hz, H-17); ^13^C NMR (150 MHz, CDCl_3_): *δ*_C_ 41.5 (C-1), 18.6 (C-2), 39.3 (C-3), 33.7 (C-4), 53.7 (C-5), 20.9 (C-6), 35.9 (C-7), 67.6 (C-8), 48.0 (C-9), 39.5 (C-10), 62.1 (C-11), 85.5 (C-12), 154.7 (C-13), 55.5 (C-14), 128.5 (C-15), 167.7 (C-16), 55.2 (C-17), 33.7 (C-18), 21.5 (C-19), 15.3 (C-20), 22.0 (C-1’), 170.7 (C-2’).

Jolkinolide E (**8**): white amorphous powder, HRESIMS *m*/*z* 301.2160 [M + H]^+^ (calcd. for C_20_H_29_O_2_, 301.2162); ^1^H NMR (600 MHz, CDCl_3_): *δ*_H_ 0.85 (3H, s, Me-18), 0.91 (3H, s, Me-17), 0.92 (3H, s, Me-19), 1.82 (3H, s, Me-20), 2.20 (1H, br d, *J* = 8.0 Hz, H-9), 2.50 (1H, dm, *J* = 13.4 Hz, H-7), 2.57 (1H, dd, *J* = 6.2, 13.5 Hz, H-11), 4.88 (1H, dd, *J* = 6.0, 13.4 Hz, H-12); ^13^C NMR (150 MHz, CDCl_3_): *δ*_C_ 39.9 (C-1), 19.3 (C-2), 42.1 (C-3), 33.8 (C-4), 55.5 (C-5), 24.1 (C-6), 37.4 (C-7), 156.5 (C-8), 52.1 (C-9), 41.8 (C-10), 27.7 (C-11), 76.3 (C-12), 152.6 (C-13), 114.1 (C-14), 116.4 (C-15), 175.7 (C-16), 8.5 (C-17), 33.8 (C-18), 22.0 (C-19), 17.0 (C-20).

Euphopilolide (**9**): white amorphous powder, HRESIMS *m*/*z* 317.2107 [M + H]^+^ (calcd. for C_20_H_29_O_3_, 317.2111); ^1^H NMR (600 MHz, CDCl_3_): *δ*_H_ 0.89 (3H, s, H-19), 0.93 (3H, s, H-18), 1.06 (3H, s, H-20), 1.97 (3H, s, H-17), 2.29 (1H, dd, *J* = 5.6, 13.4 Hz, H-11), 3.76 (1H, s, H-14); ^13^C NMR (150 MHz, CDCl_3_): *δ*_C_ 41.6 (C-1), 18.5 (C-2), 41.1 (C-3), 33.5 (C-4), 54.3 (C-5), 21.2 (C-6), 35.0 (C-7), 61.3 (C-8), 49.4 (C-9), 39.5 (C-10), 24.0 (C-11), 75.8 (C-12), 156.0 (C-13), 56.4 (C-14), 128.8 (C-15), 174.3 (C-16), 9.0 (C-17), 34.2 (C-18), 22.3 (C-19), 19.5 (C-20).

Ent-abieta-8, 11, 13-triene-7-one (**10**): yellowish oil, HRESIMS *m*/*z* 285.2214 [M + H]^+^ (calcd. for C_20_H_29_O, 285.2213); ^1^H NMR (600 MHz, CDCl_3_): *δ*_H_ 0.93 (3H, s, Me-18), 1.00 (3H, s, Me-19), 1.23 (3H, s, Me-20), 1.24 (3H, d, *J* = 6.9 Hz, Me-16), 1.24 (3H, d, *J* = 6.9 Hz, Me-17), 1.88 (1H, dd, *J* = 13.9, 4.0 Hz, H-5), 2.64 (1H, dd, *J* = 18. 1, 13. 9 Hz, H-6β), 2.73 (1H, dd, *J* = 18.2, 4.0, H-6α), 2.92 (1H, sept, *J* = 6.8 Hz, H-15), 7.29 (1H, d, *J* = 8.1 Hz, H-11), 7.39 (1H, dd, *J* = 8.1, 2.1 Hz, H-12), 7.87 (1H, d, *J* = 2.0 Hz, H-14), 1.24 (3H, d, *J* = 6.9 Hz, Me-16); ^13^C NMR (150 MHz, CDCl_3_): *δ*_C_ (C-1), 19.0 (C-2), 41.5 (C-3), 33.4 (C-4), 49.5 (C-5), 36.4 (C-6), 200.2 (C-7), 130.8 (C-8), 154.0 (C-9), 38.0 (C-10), 123.9 (C-11), 132.7 (C-12), 146.8 (C-13), 125.0 (C-14), 33.7 (C-15), 23.9 (C-16), 24.0 (C-17), 32.7 (C-18), 21.5 (C-19), 23.5 (C-20).

6*β*-Hydroxy-ent-abieta-8,11,13-triene (**11**): yellowish oil, HRESIMS *m*/*z* 287.2363 [M + H]^+^ (calcd. for C_20_H_31_O, 287.2369); ^1^H NMR (600 MHz, CDCl_3_): *δ*_H_ 0.94 (3H, s, Me-19), 0.98 (3H, s, Me-18), 1.13 (3H, s, Me-20), 1.24 (3H, d, *J* = 6.9 Hz, Me-16), 1.24 (3H, d, *J* = 6.9 Hz, Me-17), 2.87 (1H, sept, *J* = 7.0 Hz, H-15), 4.82 (1H, br s, H-6α), 7.12 (1H, dd, *J* = 8.2, 2.0 Hz, H-12), 7.20 (1H, d, *J* = 1.9 Hz, H-14), 7.21 (1H, d, *J* = 8.3 Hz, H-11); ^13^C NMR (150 MHz, CDCl_3_): *δ*_C_ 38.6 (C-1), 19.4 (C-2), 41.7 (C-3), 33.1 (C-4), 44.8 (C-5), 68.5 (C-6), 28.8 (C-7), 136.2 (C-8), 147.6 (C-9), 38.0 (C-10), 38.0 (C-11), 124.6 (C-12), 146.5 (C-13), 127.9 (C-14), 33.1 (C-15), 24.2 (C-16), 24.2 (C-17), 33.3 (C-18), 21.8 (C-19), 24.0 (C-20).

### 3.4. Cytotoxic Activity

All breast cell lines (MCF-10A, MCF-7, ZR-75-1 and MDA-MB-231) were purchased from National Collection of Authenticated Cell Cultures. The normal mammary epithelial cell line (MCF-10A) was cultured using Mammary Epithelial Cell Growth Medium BulletKit^TM^ (Clonetics, Lot No. 0001052347). MCF-7 cell line was cultured in MEM medium (GIBCO, Lot No. 41500034) with NaHCO_3_ 1.5 g/L, sodium pyruvate 0.11 g/L and 0.01 mg/mL bovine insulin. ZR-75-1 cell line was cultured in RPMI-1640 medium (GIBCO, Lot No. C11875500BT) with 10% FBS. The three cell lines were maintained in a humidified atmosphere with 5% CO_2_ at 37 °C. MDA-MB-231 cell line was cultured with L-15 medium (GIBCO, Lot No. 41300039) containing 10% FBS in a humidified atmosphere at 37 °C without CO_2_.

The cytotoxic effects of compounds **1**–**11** (HPLC, purities > 95%) on MCF-10A, MCF-7, ZR-75-1 and MDA-MB-231 cell lines were evaluated by MTT method [23]. The stock solutions (100 mg/mL) of these 11 compounds were separately prepared in Dimethyl sulfoxide (DMSO). Then the stock solutions were diluted by medium. The cells (1 × 10^4^ cells/well) were seeded in 96-well plates and cultured for about 15 h. After that, the cells were treated with compounds at different concentrations (100 μg/mL, 50 μg/mL, 25 μg/mL, 12.5 μg/mL, 6.25 μg/mL, 3.125 μg/mL) for 24 h at 37 °C with 5% CO_2_ or 100% air. After incubation, 20 μL MTT solution (5 mg/mL) was added and incubated for another 4 h. Finally, the supernatant was discarded and 150 μL DMSO was added to dissolve the purple crystal. The absorbance of the samples was read at 570 nm on an ELISA plate reader. The inhibition was expressed as IC_50_ value, which stands for inhibition of cell growth by 50%. The data is presented as the mean of three independent tests in which each compound concentration was experimented in three replicate wells and analyzed using SPSS 22.0.

### 3.5. Chromatographic Conditions

UPLC system (Waters, Milford, MA, USA) consisted of PDA eλ detector, sample manager and quaternary solvent manager with a ACQUITY UPLC^®^BEH C_18_ (50 mm × 2.1 mm, 1.7 μm). Mobile phases were water with methanol (A) and water (B). The isocratic elution was as follows: 0.01–3.00 min, 70% A. The injection volume of sample was 2 μL. The flow rate was 0.4 mL·min^−1^ and the column temperature was 35 °C. 17-Hydroxyjolkinolide B and jolkinolide B were detected at the wavelength of 240 nm. 17-Hydroxyjolkinolide A and jolkinolide A were detected at the wavelength of 285 nm.

### 3.6. Extraction Procedure

A total of 0.1 g of the samples, 3.3 g salt, 5.0 mL acetonitrile and 5.0 mL water were added into a 15 mL centrifuge tube. To obtain a good extraction efficiency of the target compounds, several experimental parameters were discussed. The type and dosage of salt were discussed. Different solvents and acetonitrile to water ratio were considered to be optimized. The impact of pH of water and weight of samples were evaluated. Then the centrifuge tubes thoroughly shaken by vortex and the effect of vortex time was studied. Subsequently, the tubes were placed into a centrifuge at 14,000 rpm for 10 min. The supernatant liquor was collected and a volume of 2 μL sample was injected into UPLC system.

### 3.7. Method Validation

The repeatability was evaluated by six parallel extracts of *E. fischeriana*. RSDs were calculated to evaluate the precision. Recovery was examined by using spiked samples of *E. fischeriana*.

### 3.8. Responses Surface Methodology

RSM was employed to determine the optimum levels of dosage of salt (*X*_1_), pH of solvent (*X*_2_), and acetonitrile-to-water ratio (*X*_3_) related to responses yields of the contents of four diterpenoids. Moreover, BBD with RSM was applied to identify the best combination of the parameters. The effect of three parameters on the extractions were investigated at three levels (−1, 0 and +1). In total, 17 experiments were conducted in random order. The values were fitted with a second-order polynomial model given below:

$$Y=\beta_{0}+\overset{3}{\underset{i=1}{\sum }}\beta_{i}X_{i}+\overset{3}{\underset{i=1}{\sum }}\beta_{ii}X_{i}^{2}+\overset{3}{\underset{i=1}{\sum }}\overset{3}{\underset{j=i+1}{\sum }}\beta_{ij}X_{i}X_{j}$$

where *Y* was the response; *Xi* and *Xj* were the independent variables influencing the response *Y*; *β*_0_, *β_i_*, *β_ii_*, and *β_ij_* described the regression coefficients for intercept, linear, quadratic and interaction terms, respectively. Design-Expert 8.0.6 was used to statistically analyze the data. The quality of fit of the polynomial model was evaluated with respect to the coefficient of determination (*R*^2^) and F-test. The lack of fit F-value (*p* < 0.05) was acquired by analysis of variance (ANOVA) and used to demonstrate variable significance and model adequacy.

### 3.9. Ultrasonic Extraction

In order to compare the SALLE with the conventional ultrasonic-assisted extraction, 0.1 g crushed sample was precisely weighed and introduced into a 15 mL centrifuge tube, then mixed with 10 mL acetonitrile. Finally, the mixture was extracted ultrasonically for 1.0 h. The extract solution was prepared by centrifugation at 14,000 rpm for 10 min. 2 μL of the supernatant liquor was injected into the UPLC system for further analysis.

## 4. Conclusions

In this study, 11 ent-abietane diterpenoids (**1**–**11**) were isolated from *E. fischeriana*. Among them, compound **1** was a novel compound, compound **10** was isolated from *Euphorbia* genus for the first time, compound **11** was discovered from *E. fischeriana* for the first time. The cytotoxic activity of the isolated compounds was tested. The results suggested that the acetoxyl group or hydroxyl group at C-17 and the epoxy group at C11-C12 were important for the activity. RSM with BBD was used to study SALLE of ent-abietane diterpenoids, which 17-hydroxyjolkinolide B, jolkinolide B, 17-hydroxyjolkinolide A and jolkinolide A were selected as quality control. The experimental values of 17-hydroxyjolkinolide B, jolkinolide B, 17-hydroxyjolkinolide A and jolkinolide A (2.134, 0.529, 0.396, and 0.148 mg/g, respectively) agreed with those predicted (2.134, 0.529, 0.396, and 0.148 mg/g, respectively) by RSM models, thus demonstrating the fitness of the model employed and the accomplishment of RSM in optimizing the extraction conditions.

## Figures and Tables

**Table 1:** ^1^H and ^13^C NMR spectroscopic data of compound **1**.

Position	1	
	*δ*_H_	*δ*_C_
1	1.32, 2.04 (o ^a^, each 1H)	44.9
2	4.93 (m, 1H)	68.0
3	1.31, 1.84 (o, each 1H)	46.4
4	1.24 (o, 1H)	35.0
5		53.1
6	1.59, 1.83 (m, each 1H)	20.6
7	1.65, 2.15 (m, each 1H)	34.1
8		61.0
9	2.67 (d, *J* = 5.10 Hz, 1H)	51.8
10		42.7
11	5.42 (d, *J* = 5.34 Hz, 1H)	103.1
12		148.0
13		144.8
14	3.72 (s, 1H)	54.6
15		126.0
16		170.5
17	2.07 (s, 3H)	8.9
18	1.02 (s, 3H)	33.6
19	0.96 (s, 3H)	22.8
20	0.83 (s, 3H)	103.6
1′		170.6
2′	2.04 (s, 3H)	21.6

^a^ overlapped resonances.

**Table 2:** In vitro cytotoxicity of **1**–**11** against MCF-10A, MCF-7, ZR-75-1 and MDA-MB-231 cell lines (*μ*g·mL^−1^, Means ± S.D., *n* = 3).

Compounds	IC_50_			
	MCF-10A	MCF-7	ZR-75-1	MDA-MB-231
**1**	88.8 ± 1.8	59.6 ± 2.1	225.2 ± 2.8	105.9 ± 2.1
**2**	114.6 ± 1.7	169.4 ± 2.2	104.8 ± 1.9	162.5 ± 3.2
**3**	83.3 ± 0.9	94.4 ± 1.6	73.1 ± 0.9	43.6 ± 1.6
**4**	39.4 ± 0.4	41.0 ± 0.7	44.9 ± 0.5	71.5 ± 2.5
**5**	3.4 ± 0.1	4.7 ± 0.2	2.2 ± 0.1	1.1 ± 0.1
**6**	10.4 ± 0.2	7.8 ± 0.2	3.3 ± 0.1	19.9 ± 0.3
**7**	4.3 ± 0.1	3.4 ± 0.1	1.2 ± 0.1	1.7 ± 0.1
**8**	31.6 ± 0.3	104.8 ± 2.2	127.6 ± 2.3	103.4 ± 2.4
**9**	70.2 ± 0.5	95.2 ± 1.9	121.3 ± 2.4	100.5 ±2.6
**10**	41.5 ± 0.3	100.2 ± 1.7	113.1 ± 1.4	87.7 ± 1.9
**11**	15.8 ± 0.2	63.2 ± 1.4	61.1 ± 1.1	70.0 ± 2.3
paclitaxel	8.7 ± 0.2	4.1 ± 0.1	1.4 ± 0.1	3.2 ± 0.1

**Table 3:** Regression data, precision, repeatability and recovery for the four compounds.

Analyte	Calibratio Curve	*R* ^2^	Linearity Range (μg/mL)	Precision (%)		Repeatability (%)	Recovery (%)
				Intraday RSD	Interday RSD		
17-hydroxyl jolkinolide B	*Y* = 12688479.17*X* + 20362.72	0.9998	2.03–520.00	0.45	1.21	2.20	98.1
jolkinolide B	*Y* = 15206702.47*X* + 32980.61	0.9997	1.98–507.50	0.26	1.75	2.05	96.7
17-hydroxyl jolkinolide A	*Y* = 14312048.35*X* + 16595.40	0.9999	2.04–522.50	0.35	1.44	2.00	95.1
jolkinolide A	*Y* = 15921333.86*X* + 9858.72	1.0000	2.03–520.00	0.33	1.36	4.65	104.9

**Table 4:** Experimental design applied to extraction and responses of 4 ent-abietane diterpenoids in Box-Behnken design assays.

Run	Independent Variables			Responses			
	Dosage of Salt (g)	pH	Acetonitrile to Water Ratio	*Y*_1_ (mg/g)	*Y*_2_ (mg/g)	*Y*_3_ (mg/g)	*Y*_4_ (mg/g)
1	1.5	8	6	1.972	0.470	0.365	0.128
2	0.9	8	7	1.955	0.459	0.345	0.125
3	0.9	8	7	1.997	0.465	0.338	0.128
4	0.9	8	7	1.990	0.462	0.353	0.127
5	0.9	8	7	1.952	0.450	0.329	0.123
6	0.9	10	6	1.981	0.470	0.354	0.131
7	0.9	10	8	1.978	0.451	0.333	0.120
8	0.9	6	8	1.927	0.433	0.292	0.114
9	0.3	6	7	2.023	0.460	0.326	0.126
10	1.5	8	8	2.063	0.475	0.341	0.128
11	0.9	6	6	2.019	0.480	0.363	0.135
12	0.3	10	7	1.989	0.456	0.336	0.125
13	0.9	8	7	1.996	0.462	0.348	0.127
14	0.3	8	8	1.982	0.442	0.302	0.117
15	0.3	8	6	2.099	0.512	0.371	0.144
16	1.5	6	7	1.936	0.450	0.309	0.129
17	1.5	10	7	2.018	0.466	0.352	0.126

**Table 5:** ANOVA statistics of the quadratic model for the extraction yields of jolkinolide A, jolkinolide B, 17-hydroxyjolkinolide A and 17-hydroxyjolkinolide B.

Source	*Y*_1_		*Y*_2_		*Y*_3_		*Y*_4_	
	F Value	*p*-Value Prob > F	F Value	*p*-Value Prob > F	F Value	*p*-Value Prob > F	F Value	*p*-Value Prob > F
Model	7.67	0.0068	23.06	0.0002	10.30	0.0028	17.68	0.0005
*X*_1_	3.38	0.1086	0.40	0.5466	1.66	0.2380	0.095	0.7665
*X*_2_	1.19	0.3107	1.83	0.2183	11.19	0.0123	0.16	0.7002
*X*_3_	4.71	0.0666	95.28	<0.0001	55.11	0.0001	101.85	<0.0001
*X_1_X*_2_	8.50	0.0225	4.07	0.0834	3.61	0.0992	0.22	0.6501
*X_1_X*_3_	27.53	0.0012	62.37	<0.0001	6.58	0.0372	41.69	0.0003
*X_2_X*_3_	4.99	0.0607	8.54	0.0222	8.15	0.0245	6.01	0.0441
*X_1_^2^*	11.64	0.0113	9.92	0.0162	0.082	0.7829	5.51	0.0513
*X_2_^2^*	4.10	0.0825	14.86	0.0063	5.83	0.0465	3.57	0.1009
*X_3_^2^*	3.40	0.1078	11.64	0.0113	0.55	0.4808	0.39	0.5536
Lack of fit	0.50	0.6997	0.13	0.9366	0.72	0.5887	1.62	0.3181

## Data Availability

The data that support the findings of this study are available from the corresponding author upon reasonable request.

## Supplementary Materials

The following supporting information can be downloaded at: https://www.mdpi.com/article/10.3390/molecules27134282/s1. Figure S1: ^1^H-NMR spectrum of compound **1**; Figure S2: ^13^C-NMR spectrum of compound **1**; Figure S3: DEPT spectrum of compound **1**; Figure S4: HSQC Spectrum of compound **1**; Figure S5: HMBC spectrum of compound **1**; Figure S6: ^1^H-^1^H COSY spectrum of compound **1**; Figure S7: NOESY spectrum of compound **1**; Figure S8: HRESIMS data of compound **1**; Figure S9: The purity of compound **1**; Figure S10: ^1^H-NMR spectrum of compound **2**; Figure S11: ^13^C-NMR spectrum of compound **2**; Figure S12: HRESIMS data of compound **2**; Figure S13: The purity of compound **2**; Figure S14: ^1^H-NMR spectrum of compound **3**; Figure S15: ^13^C-NMR spectrum of compound **3**; Figure S16: HRESIMS data of compound **3**; Figure S17: The purity of compound **3**; Figure S18: ^1^H-NMR spectrum of compound **4**; Figure S19: ^13^C-NMR spectrum of compound **4**; Figure S20: HRESIMS data of compound **4**; Figure S21: The purity of compound **4**; Figure S22: ^1^H-NMR spectrum of compound **5**; Figure S23: ^13^C-NMR spectrum of compound **5**; Figure S24: HRESIMS data of compound **5**; Figure S25: The purity of compound **5**; Figure S26: ^1^H-NMR spectrum of compound **6**; Figure S27: ^13^C-NMR spectrum of compound **6**; Figure S28: HRESIMS data of compound **6**; Figure S29: The purity of compound **6**; Figure S30: ^1^H-NMR spectrum of compound **7**; Figure S31: ^13^C-NMR spectrum of compound **7**; Figure S32: HRESIMS data of compound **7**; Figure S33: The purity of compound **7**; Figure S34: ^1^H-NMR spectrum of compound **8**; Figure S35: ^13^C-NMR spectrum of compound **8**; Figure S36: HRESIMS data of compound **8**; Figure S37: The purity of compound **8**; Figure S38: ^1^H-NMR spectrum of compound **9**; Figure S39: ^13^C-NMR spectrum of compound **9**; Figure S40: HRESIMS data of compound **9**; Figure S41: The purity of compound **9**; Figure S42: ^1^H-NMR spectrum of compound **10**; Figure S43: ^13^C-NMR spectrum of compound **10**; Figure S44: HRESIMS data of compound **10**; Figure S45: The purity of compound **10**; Figure S46: ^1^H-NMR spectrum of compound **11**; Figure S47: ^13^C-NMR spectrum of compound **11**; Figure S48: HRESIMS data of compound **11**; Figure S49: The purity of compound **11**.

## Sample Availability

Not available.
