# Supplementary material for: Chemical Composition, Anti-Breast Cancer Activity and Extraction Techniques of Ent-Abietane Diterpenoids from Euphorbia fischeriana Steud
Source: Molecules. 2022 Jul 3;27(13):4282. doi: 10.3390/molecules27134282 (PMC9268248; doi:10.3390/molecules27134282)
Supplement: Supplementary file 1 [file molecules-27-04282-s001.zip › molecules-1771075-supplementary.pdf]

Supplementary Materials

Chemical Composition, Anti-Breast Cancer Activity and Extraction Techniques of Ent-Abietane Diterpenoids from *Euphorbia fischeriana* Steud

Gang Chen [1], Tiancheng Ma [1,2], Yukun Ma [1], Cuicui Han [3,*], Jinling Zhang [1] and Yu Sun [1,*]

[1]*Research Institute of Medicine and Pharmacy, Qiqihar Medical University, Bukui Road 333, Qiqihar 161006, China*

[2]*School of Traditional Chinese Materia Medica, Shenyang Pharmaceutical University, Wenhua Road 103, Shenyang 110016, China*

[3]*College of Pharmacy, Qiqihar Medical University, Bukui Road 333, Qiqihar 161006, China*

Abstract

Figure S1. ^{1}H-NMR spectrum of compound **1**.

Figure S2. ^{13}C-NMR spectrum of compound **1**.

Figure S3. DEPT spectrum of compound **1**.

Figure S4. HSQC spectrum of compound **1**.

Figure S5. HMBC spectrum of compound **1**.

Figure S6. ^{1}H-^{1}H COSY spectrum of compound **1**.

Figure S7. NOESY spectrum of compound **1**.

Figure S8. HRESIMS data of compound **1**.

Figure S9. The purity of compound **1**.

Figure S10. ^{1}H-NMR spectrum of compound **2**.

Figure S11. ^{13}C-NMR spectrum of compound **2**.

Figure S12. HRESIMS data of compound **2**.

Figure S13. The purity of compound **2**.

Figure S14. ^{1}H-NMR spectrum of compound **3**.

Figure S15. ^{13}C-NMR spectrum of compound **3**.

Figure S16. HRESIMS data of compound **3**.

Figure S17. The purity of compound **3**.

Figure S18. ^{1}H-NMR spectrum of compound **4**.

Figure S19. ^{13}C-NMR spectrum of compound **4**.

Figure S20. HRESIMS data of compound **4**.

Figure S21. The purity of compound **4**.

Figure S22. ^{1}H-NMR spectrum of compound **5**.

Figure S23. ^{13}C-NMR spectrum of compound **5**.

Figure S24. HRESIMS data of compound **5**.

Figure S25. The purity of compound **5**.

Figure S26. ^{1}H-NMR spectrum of compound **6**.

Figure S27. ^{13}C-NMR spectrum of compound **6**.

Figure S28. HRESIMS data of compound **6**.

Figure S29. The purity of compound **6**.

Figure S30. ^{1}H-NMR spectrum of compound **7**.

Figure S31. ^{13}C-NMR spectrum of compound **7**.

Figure S32. HRESIMS data of compound **7**.

Figure S33. The purity of compound **7**.

Figure S34. ^{1}H-NMR spectrum of compound **8**.

Figure S35. ^{13}C-NMR spectrum of compound **8**.

Figure S36. HRESIMS data of compound **8**.

Figure S37. The purity of compound **8**.

Figure S38. ^{1}H-NMR spectrum of compound **9**.

Figure S39. ^{13}C-NMR spectrum of compound **9**.

Figure S40. HRESIMS data of compound **9**.

Figure S41. The purity of compound **9**.

Figure S42. ^{1}H-NMR spectrum of compound **10**.

Figure S43. ^{13}C-NMR spectrum of compound **10**.

Figure S44. HRESIMS data of compound **10**.

Figure S45. The purity of compound **10**.

Figure S46. ^{1}H-NMR spectrum of compound **11**.

Figure S47. ^{13}C-NMR spectrum of compound **11**.

Figure S48. HRESIMS data of compound **11**.

Figure S49. The purity of compound **11**.

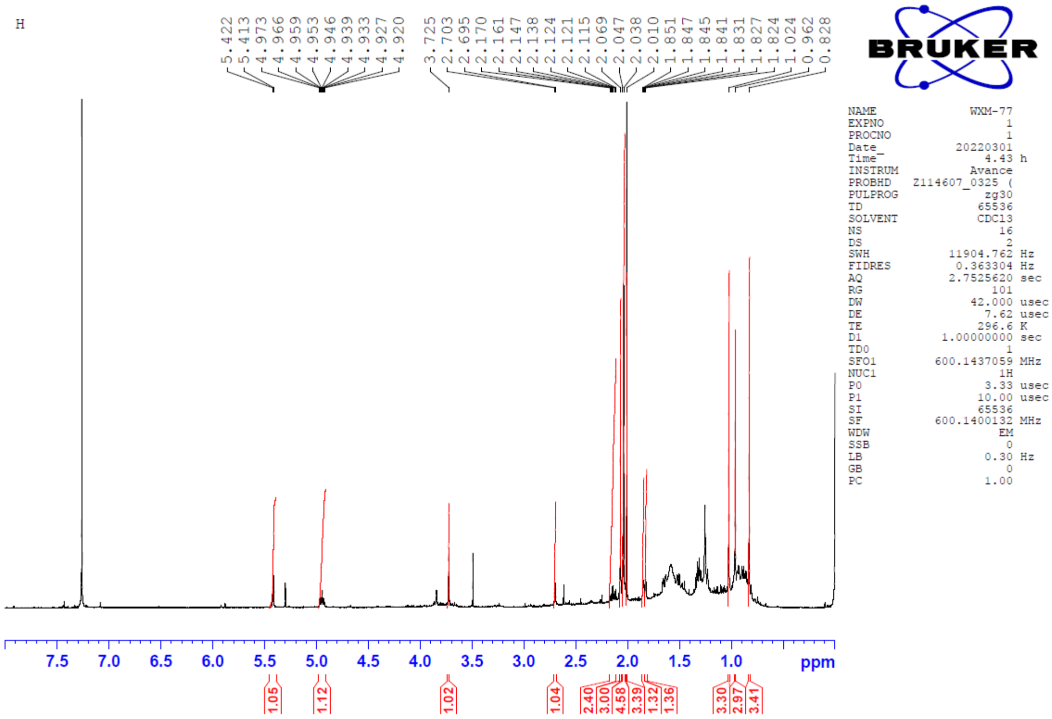

Figure S1. ^{1}H-NMR spectrum of compound **1**.

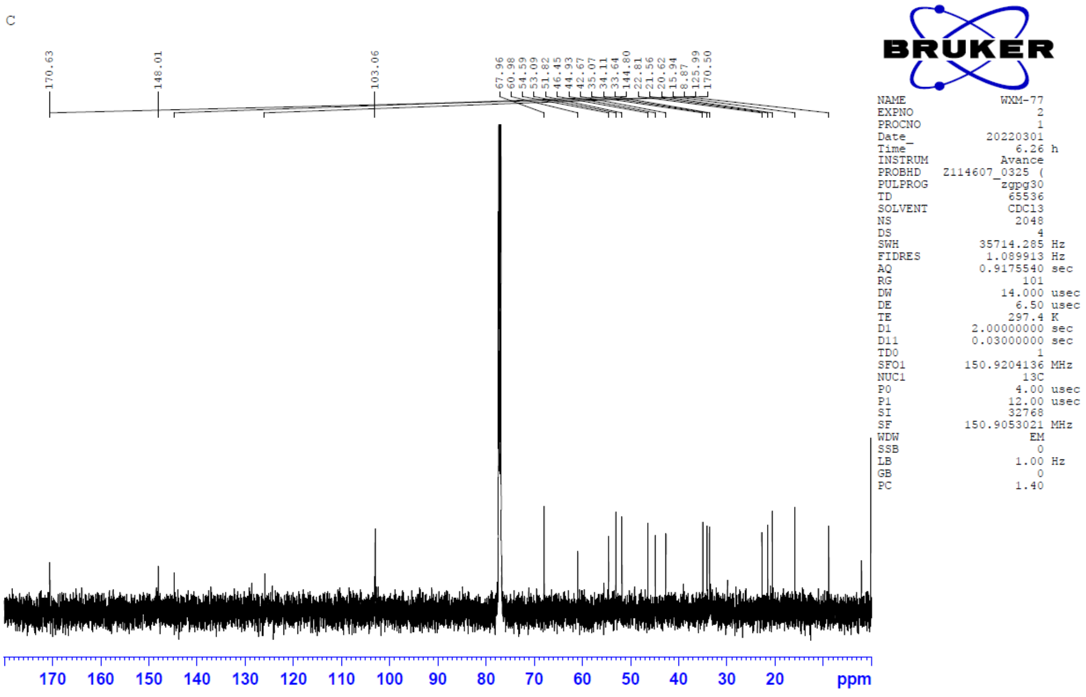

Figure S2. ^{13}C-NMR spectrum of compound **1**.

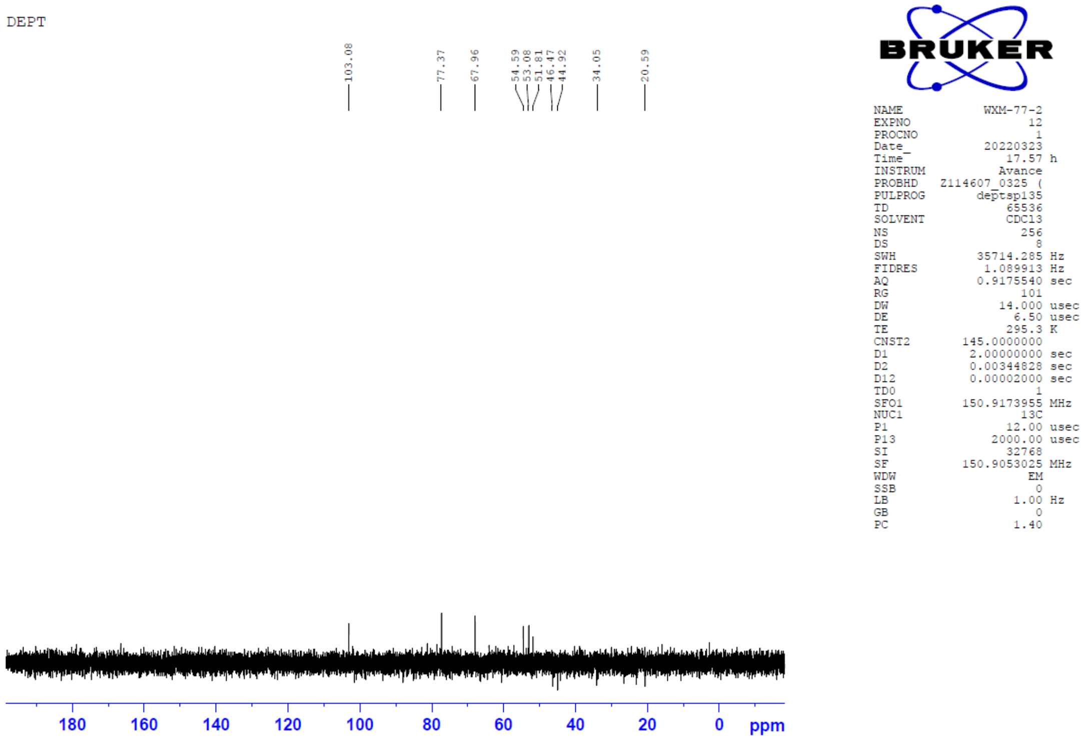

Figure S3. DEPT spectrum of compound **1**.

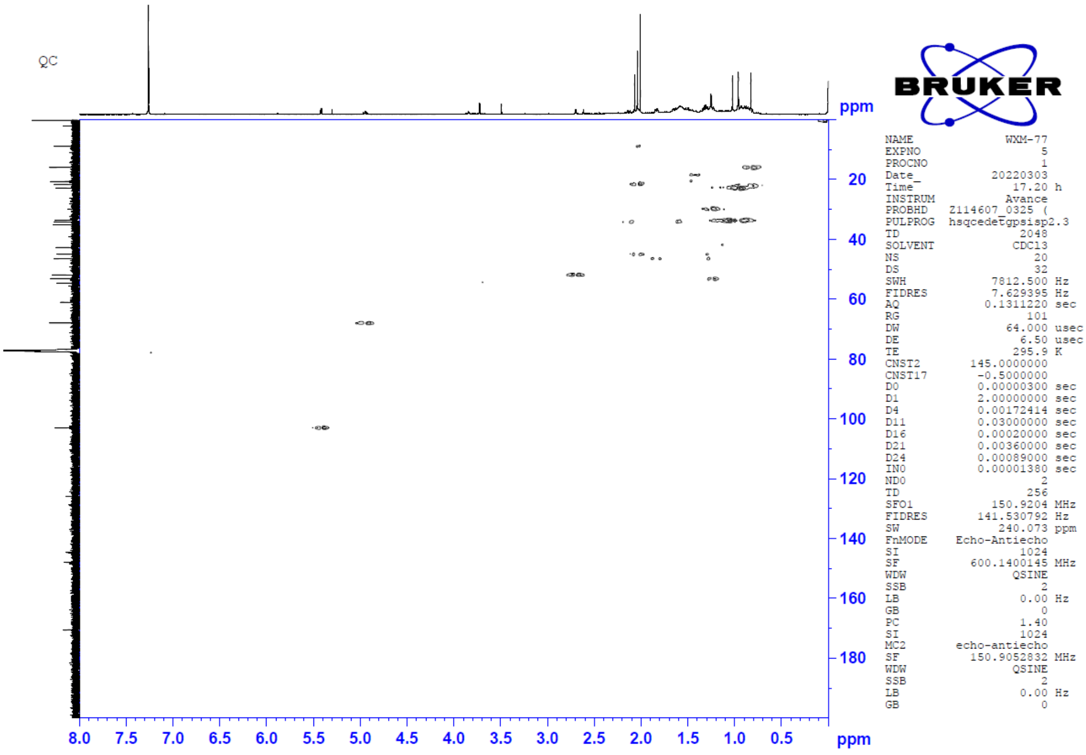

Figure S4. HSQC spectrum of compound **1**.

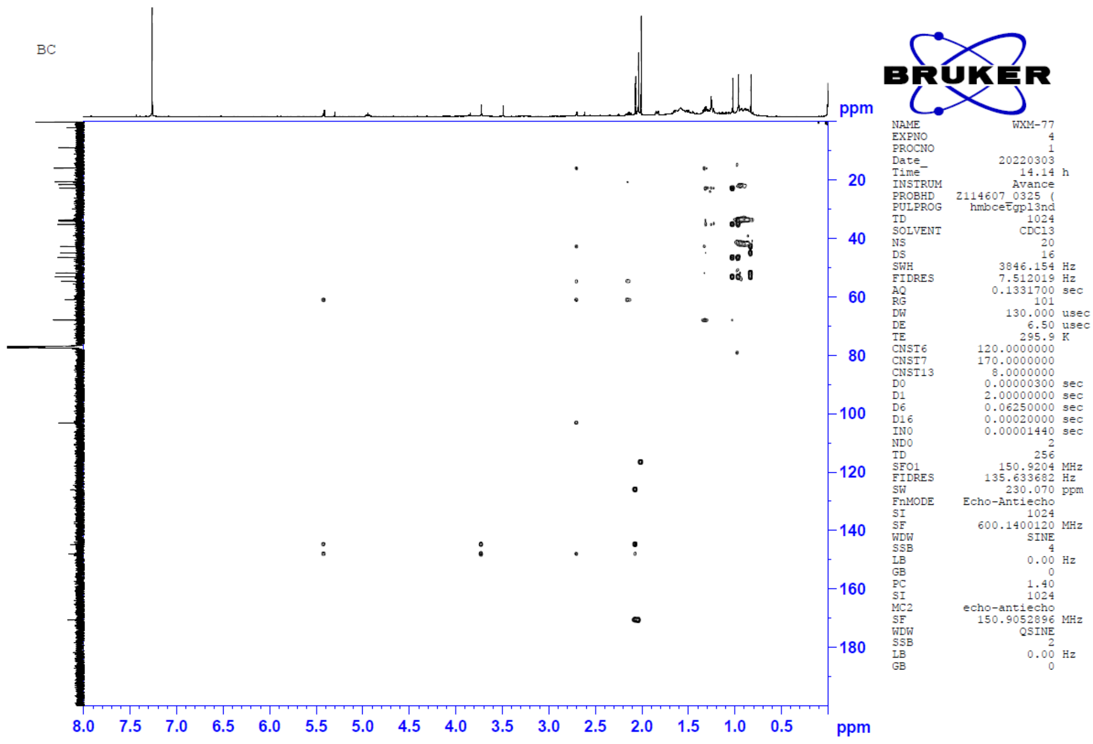

Figure S5. HMBC spectrum of compound **1**.

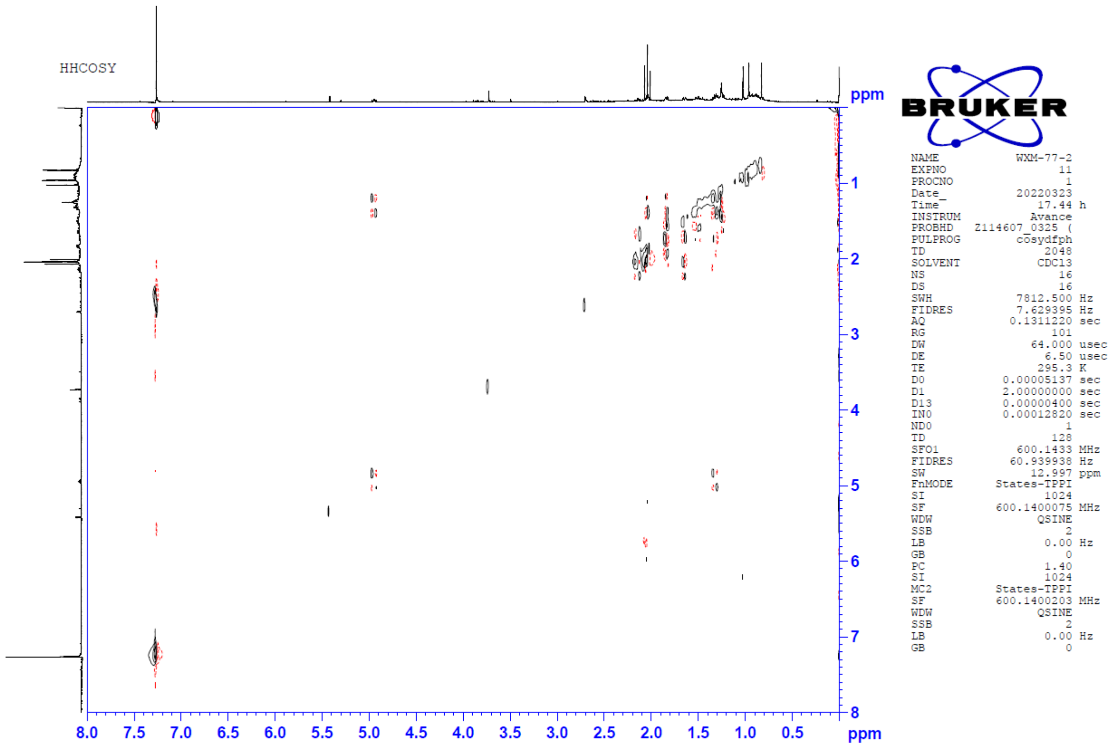

Figure S6. ^{1}H-^{1}H COSY spectrum of compound **1**.

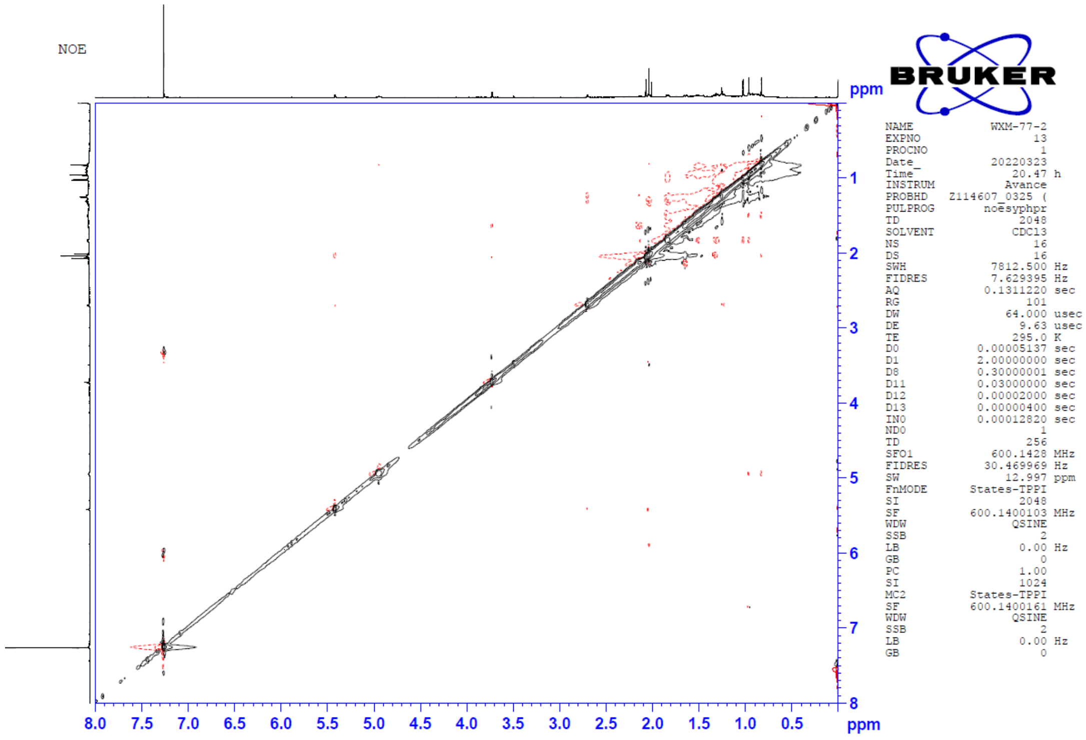

Figure S7. NOESY spectrum of compound **1**.

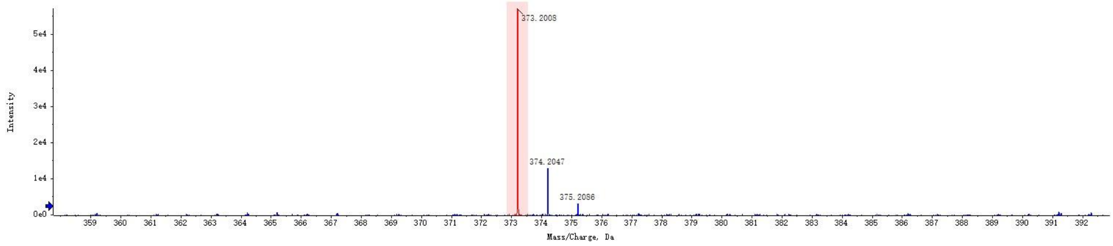

Figure S8. HRESIMS data of compound **1**.

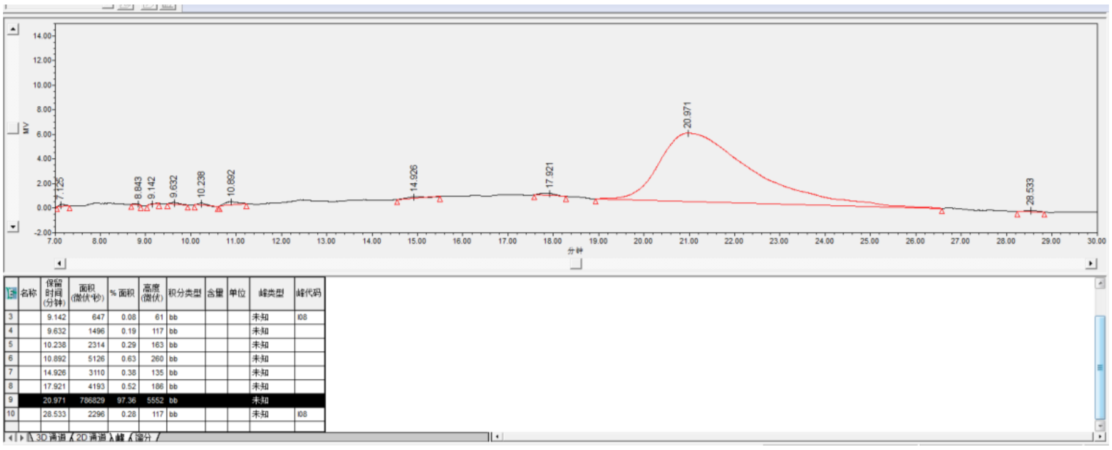

Figure S9. The purity of compound **1**.

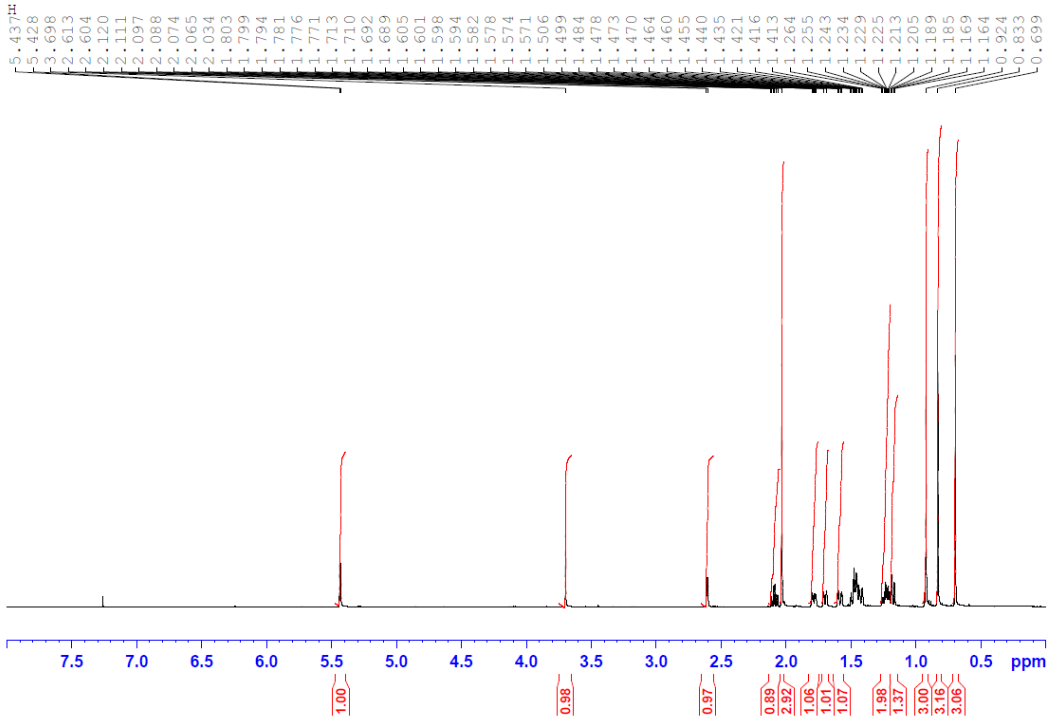

Figure S10. ^{1}H-NMR spectrum of compound **2**.

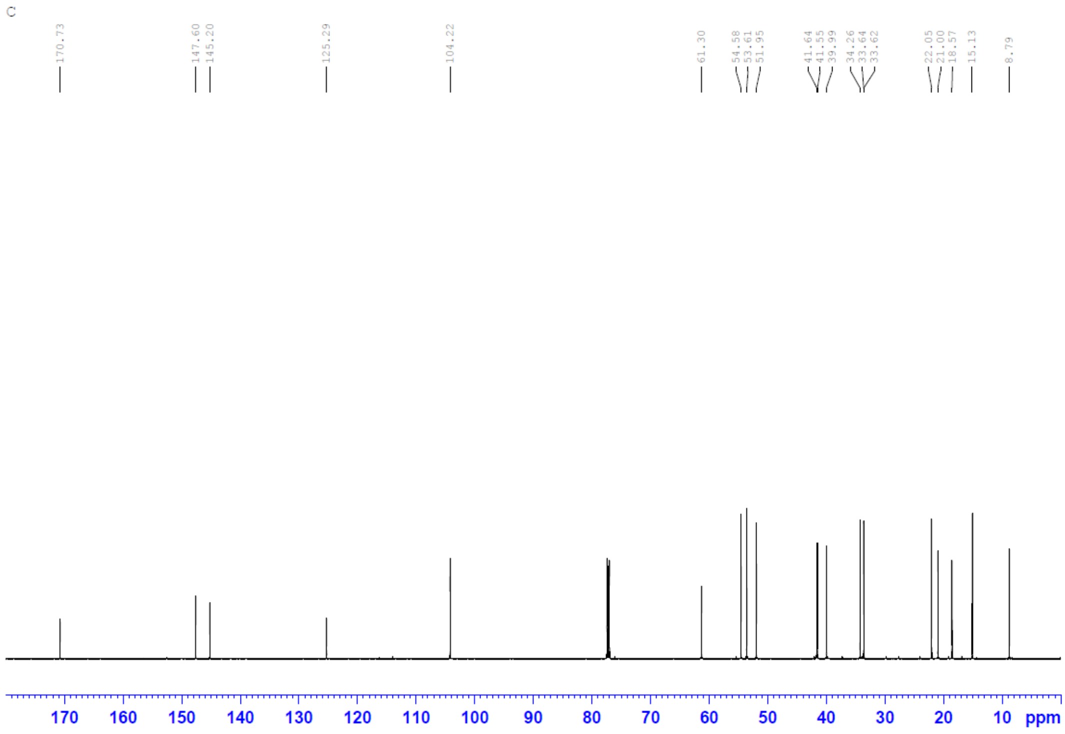

Figure S11. ^{13}C-NMR spectrum of compound **2**.

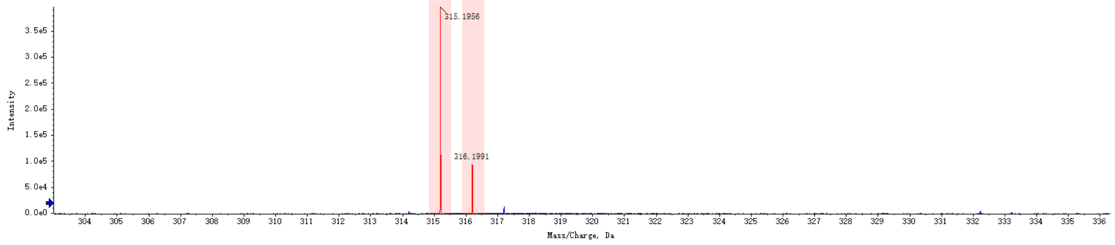

Figure S12. HRESIMS data of compound **2**.

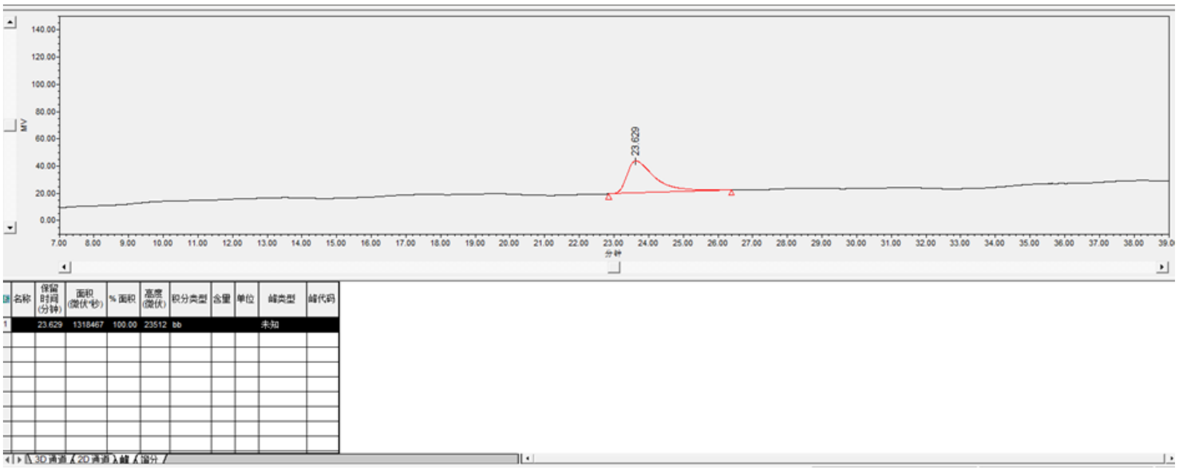

Figure S13. The purity of compound **2**.

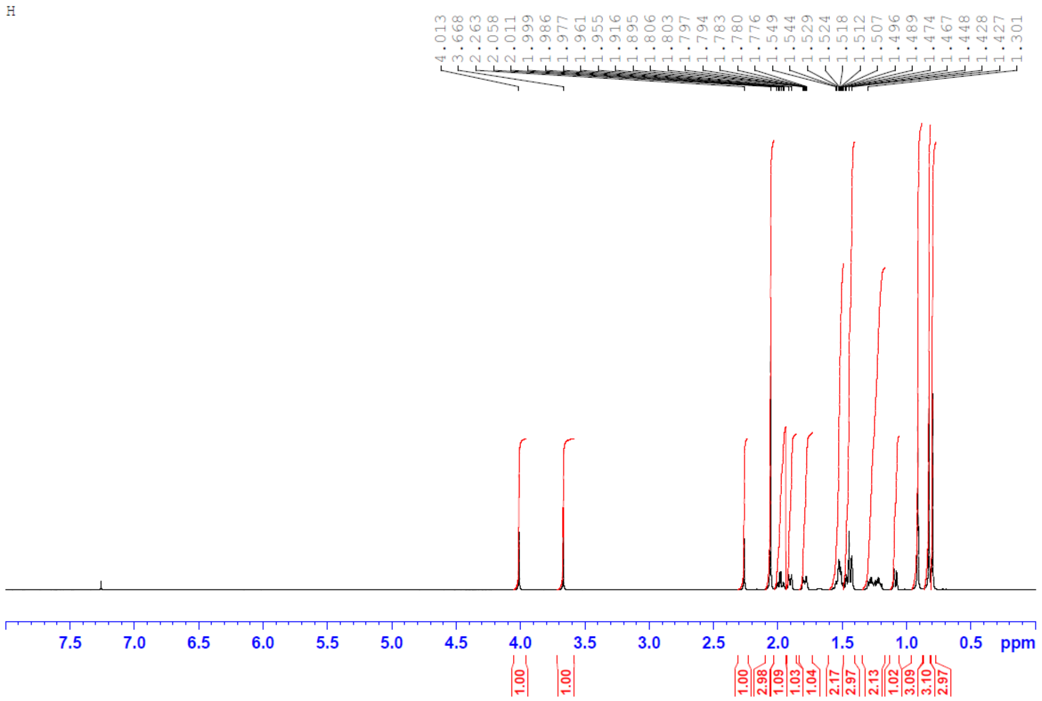

Figure S14. ^{1}H-NMR spectrum of compound **3**.

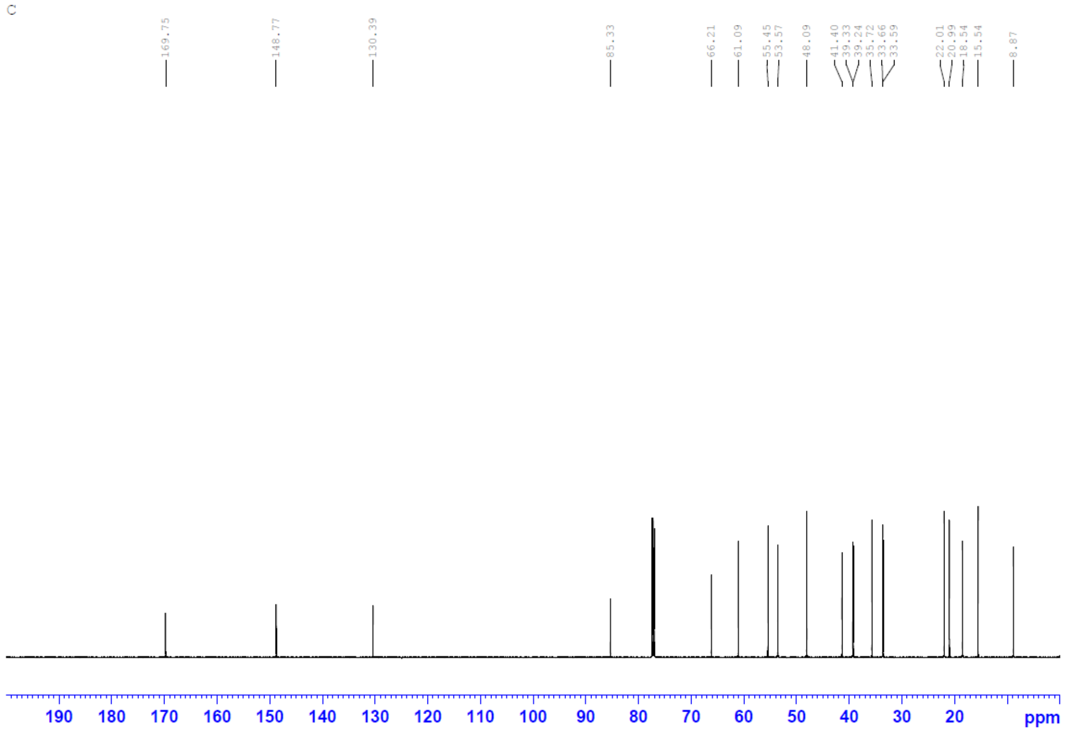

Figure S15. ^{13}C-NMR spectrum of compound **3**.

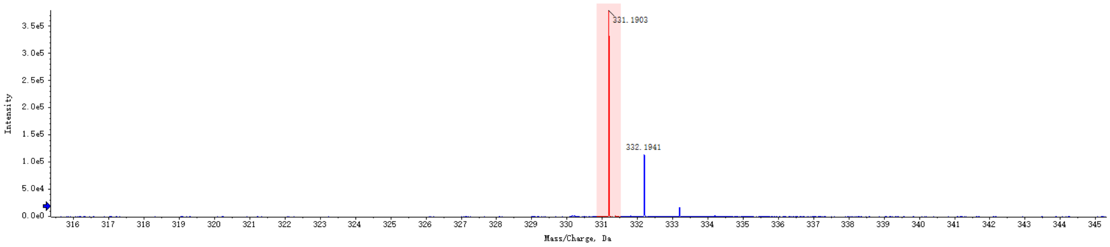

Figure S16. HRESIMS data of compound **3**.

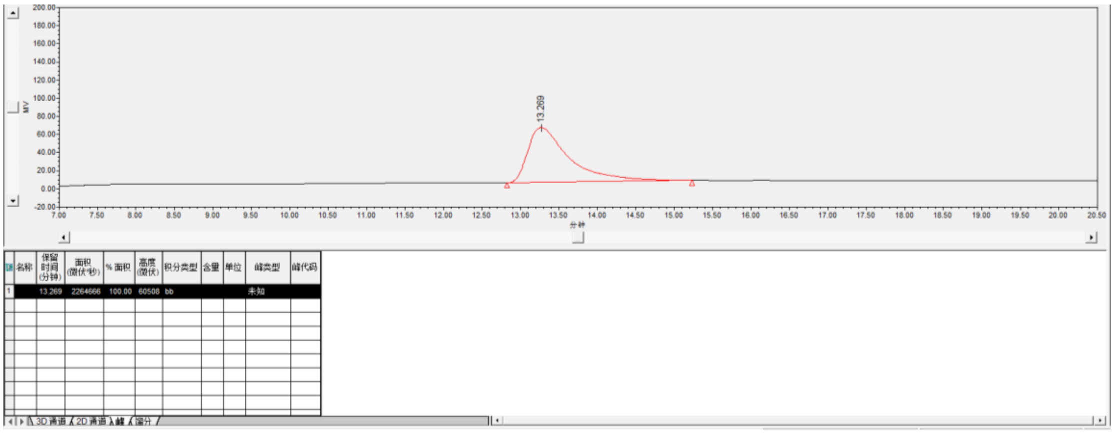

	名称	保留时间(分钟)	面积(微伏*秒)	% 面积	高度(微伏)	积分类型	含量	单位	峰类型	峰代码
1		13.269	2264666	100.00	60508	bb			未知	

Figure S17. The purity of compound **3**.

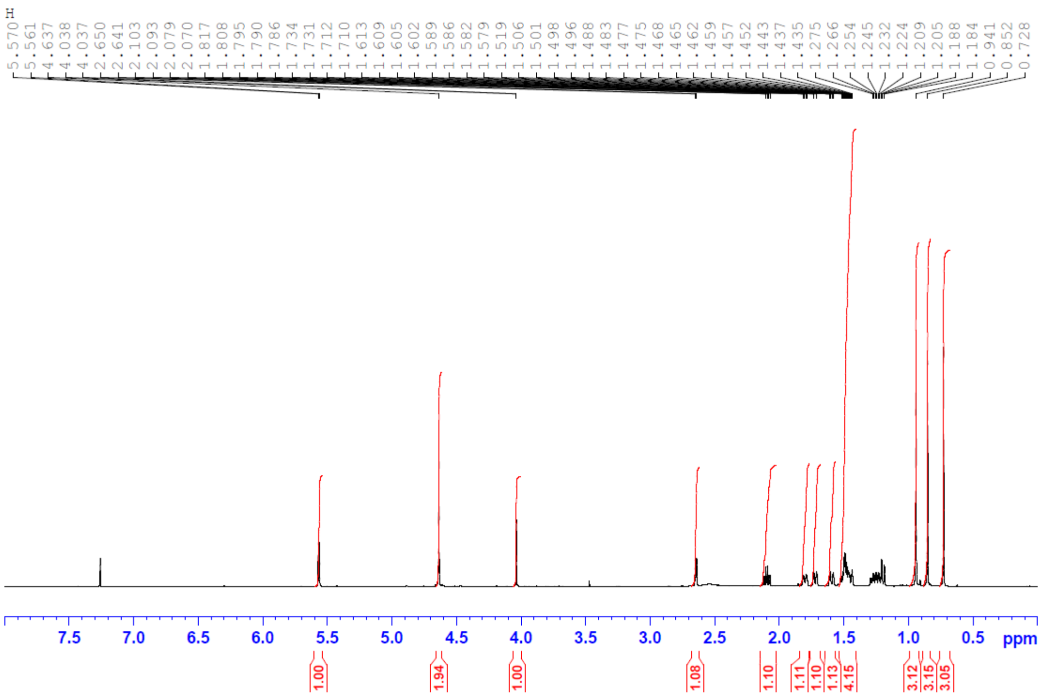

Figure S18. ^{1}H-NMR spectrum of compound **4**.

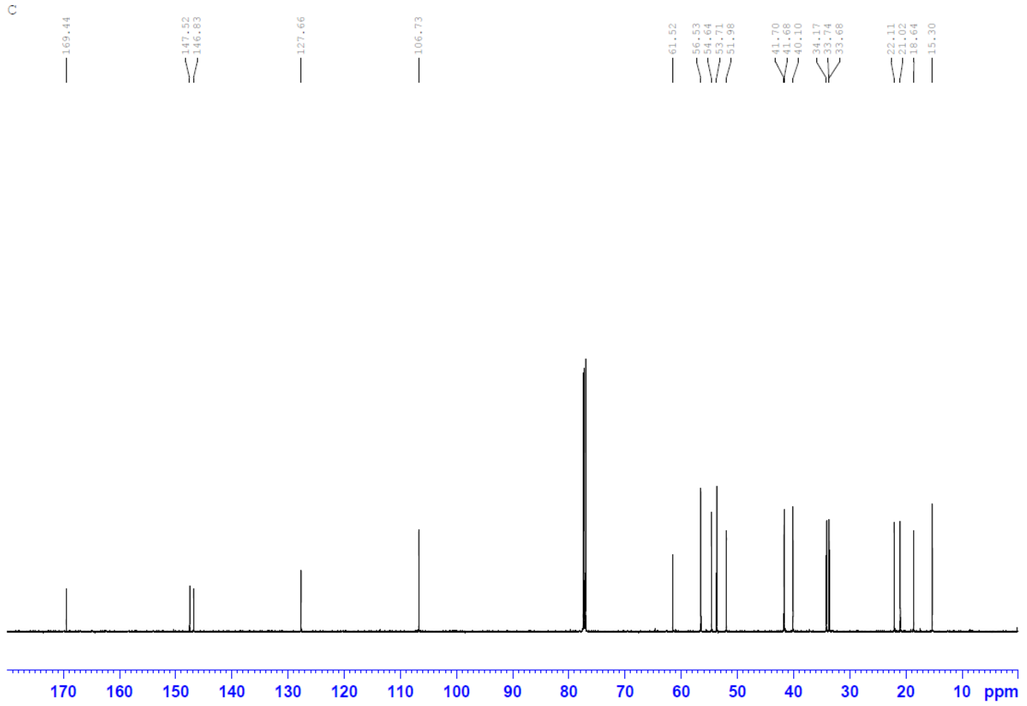

Figure S19. ^{13}C-NMR spectrum of compound **4**.

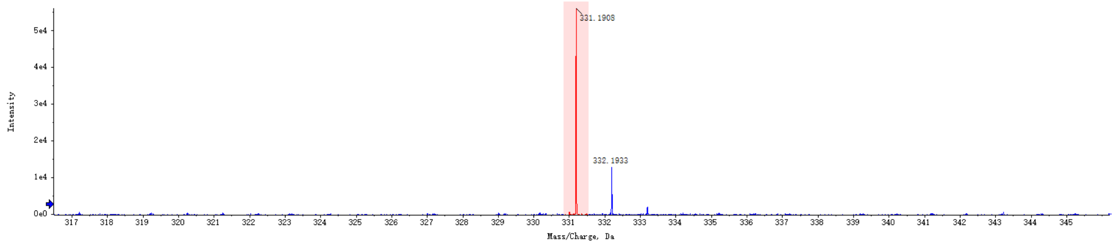

Figure S20. HRESIMS data of compound **4**.

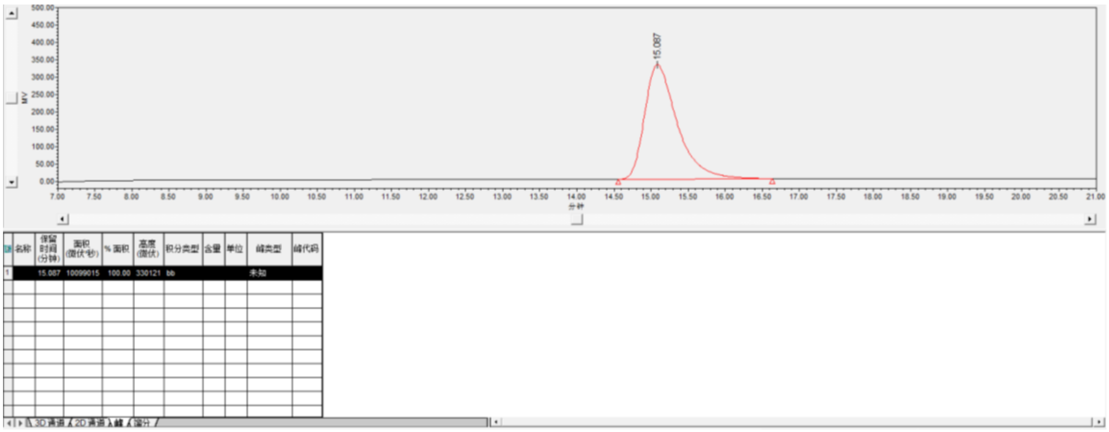

Figure S21. The purity of compound **4**.

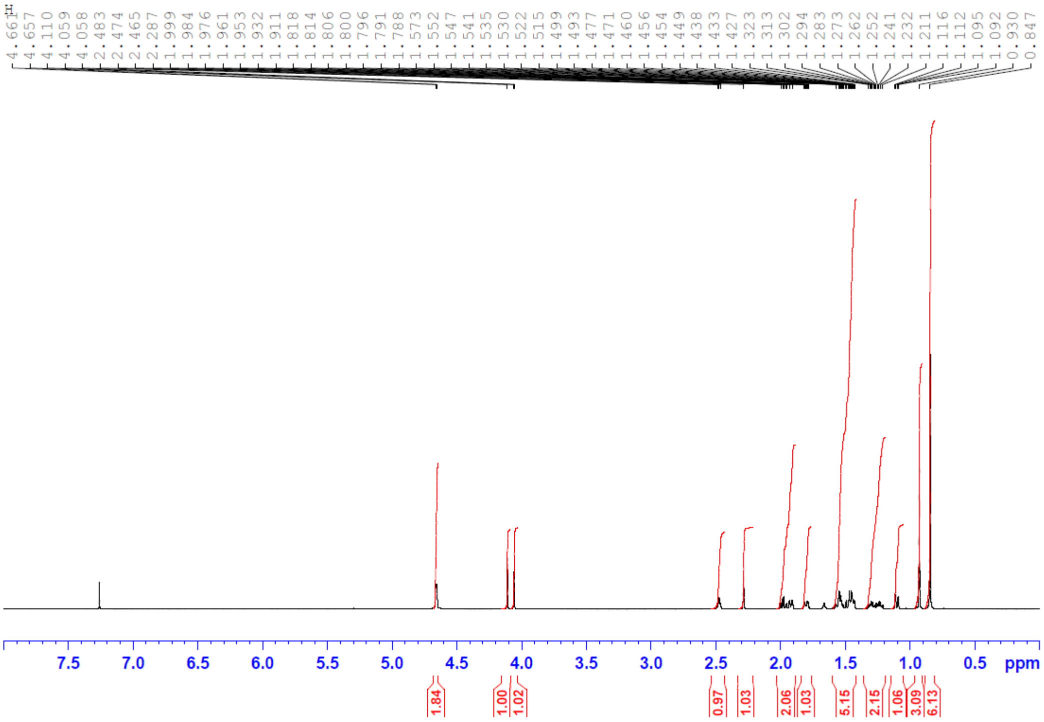

Figure S22. ^{1}H-NMR spectrum of compound **5**.

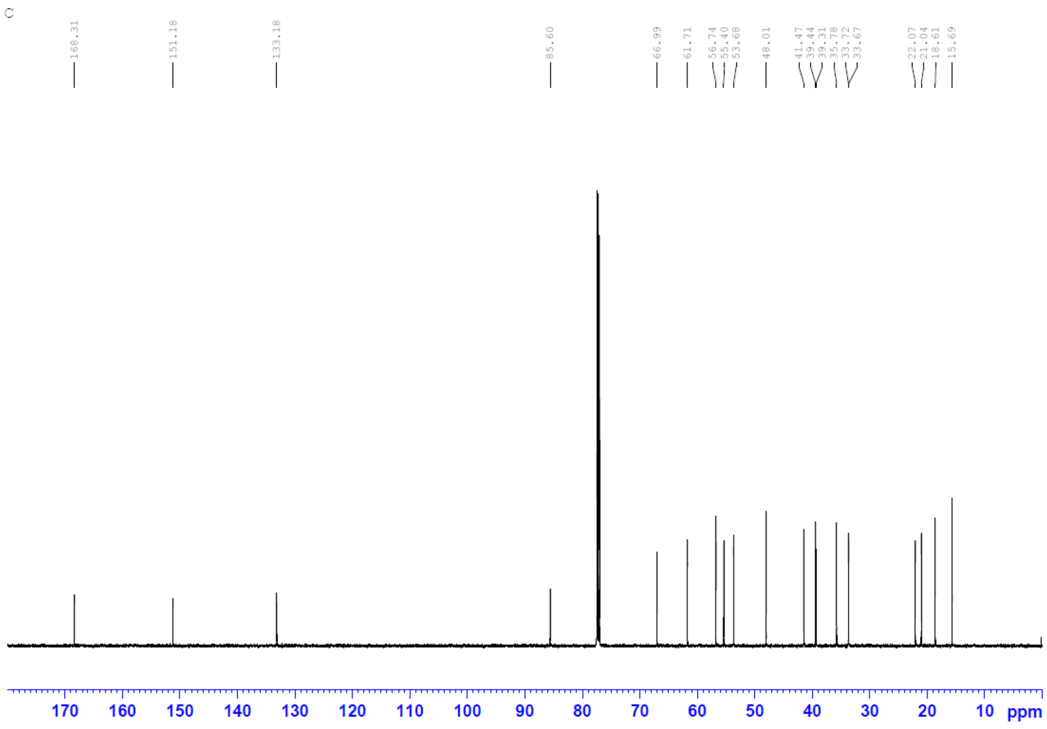

Figure S23. ^{13}C-NMR spectrum of compound **5**.

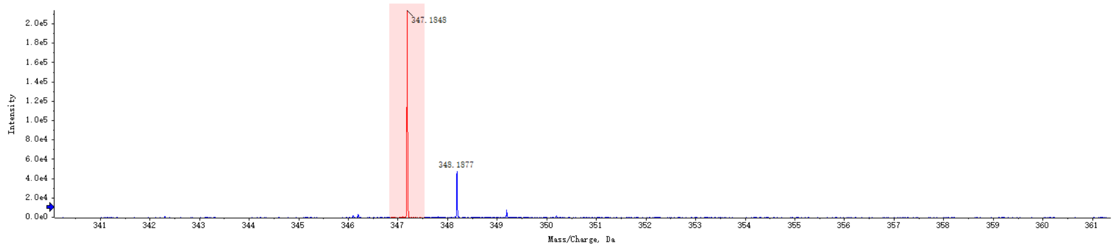

Figure S24. HRESIMS data of compound **5**.

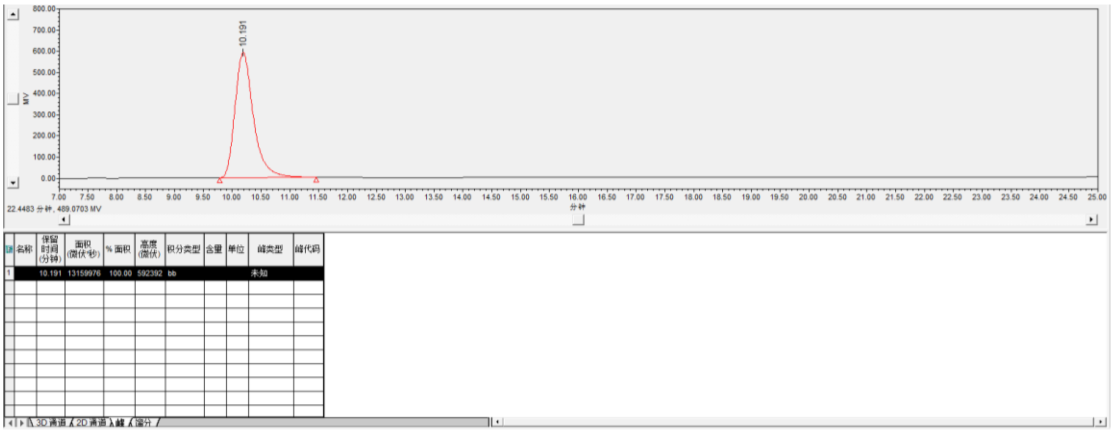

Figure S25. The purity of compound **5**.

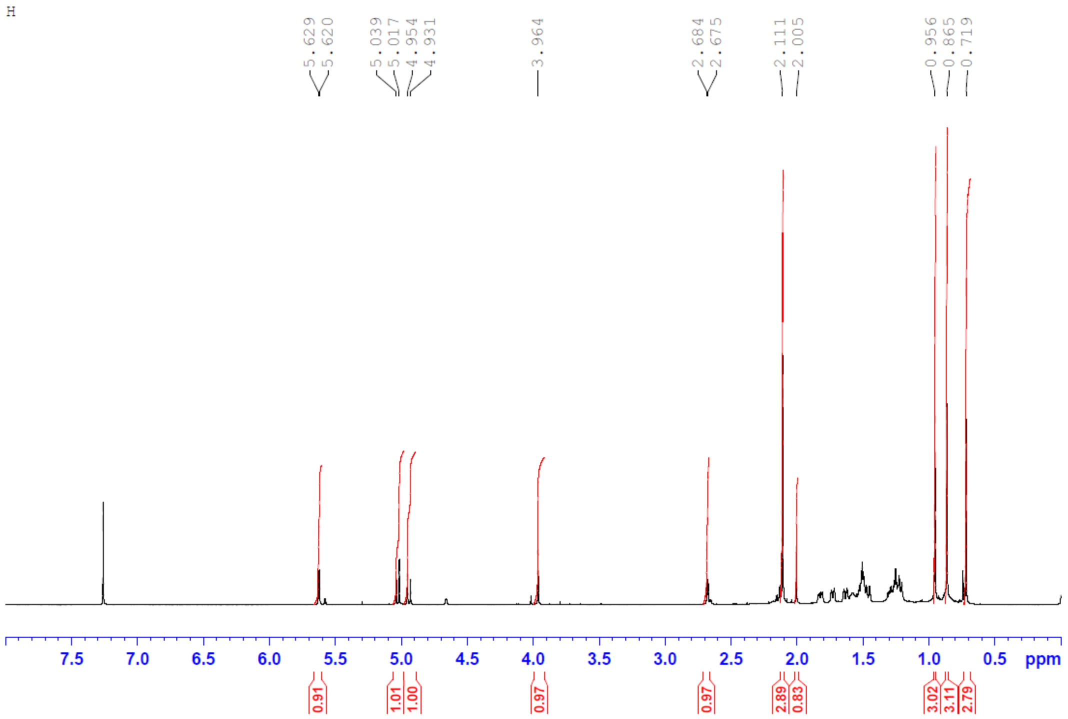

Figure S26. ^{1}H-NMR spectrum of compound **6**.

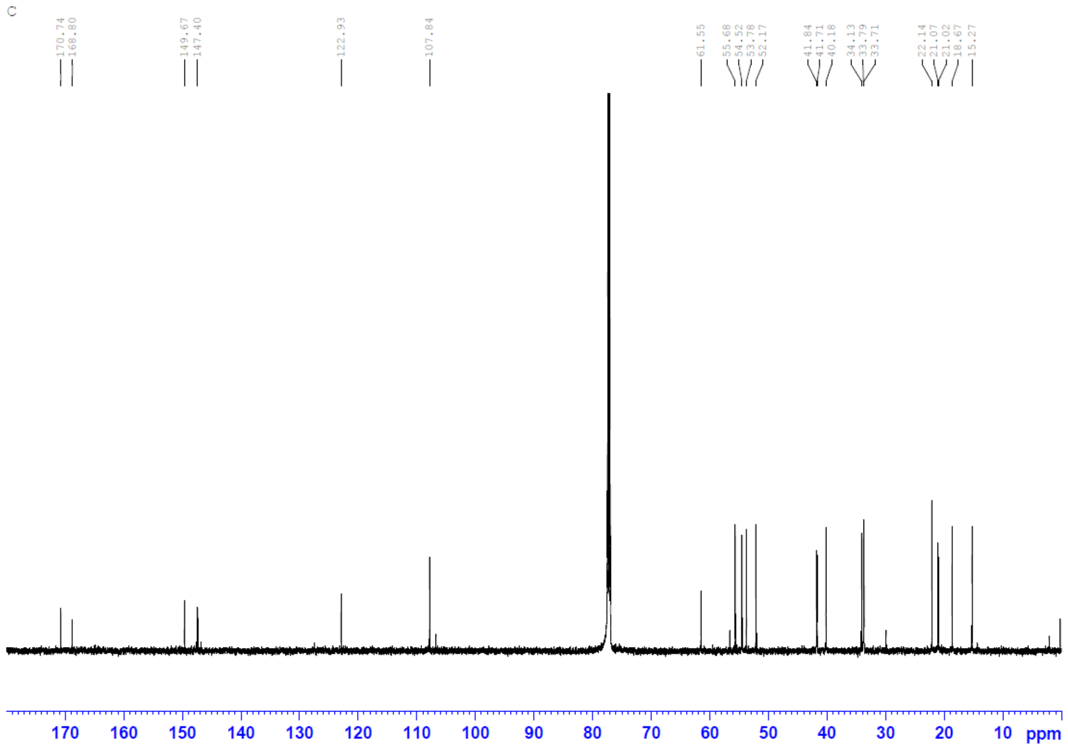

Figure S27. ^{13}C-NMR spectrum of compound **6**.

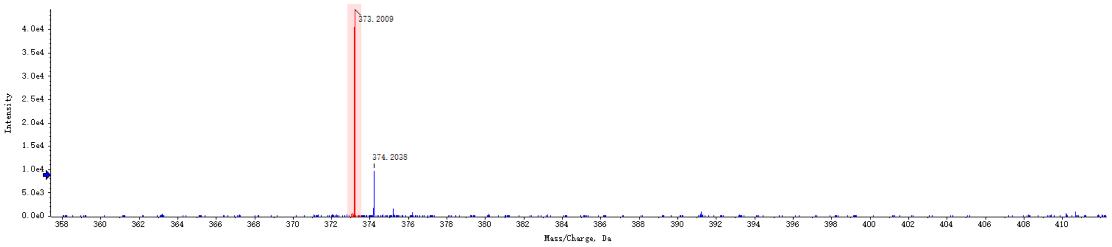

Figure S28. HRESIMS data of compound **6**.

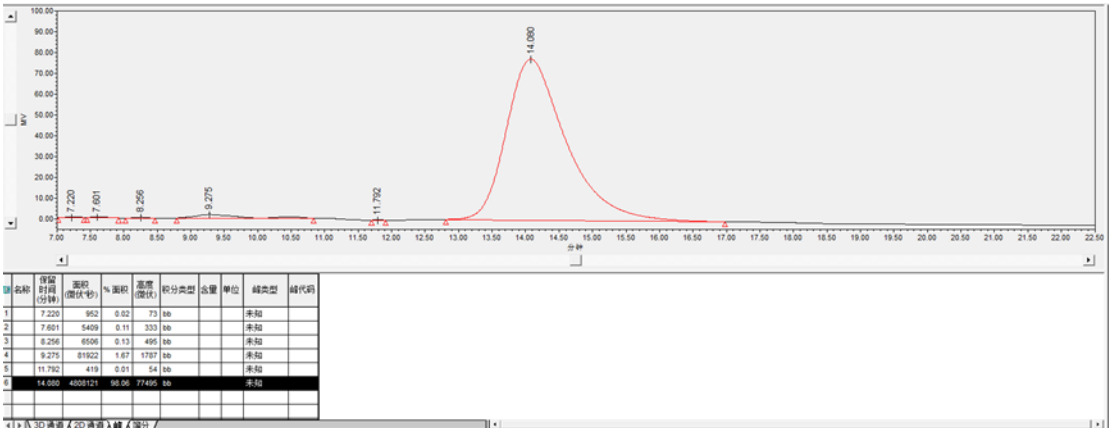

	名称	保留时间(分钟)	面积(微伏*秒)	% 面积	高度(微伏)	积分类型	含量	单位	峰类型	峰代码
1		7.220	952	0.02	73	bb			未知	
2		7.601	5409	0.11	333	bb			未知	
3		8.256	6506	0.13	495	bb			未知	
4		9.275	81922	1.67	1787	bb			未知	
5		11.792	419	0.01	54	bb			未知	
6		14.080	4808121	98.06	77495	bb			未知	

Figure S29. The purity of compound **6**.

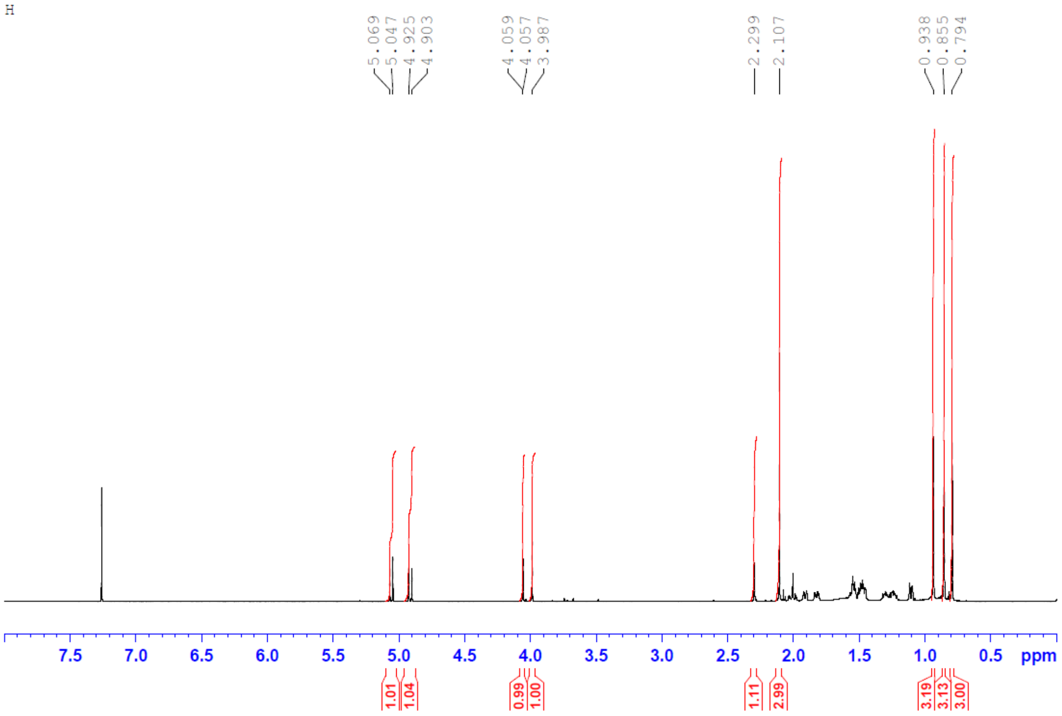

Figure S30. ^{1}H-NMR spectrum of compound **7**.

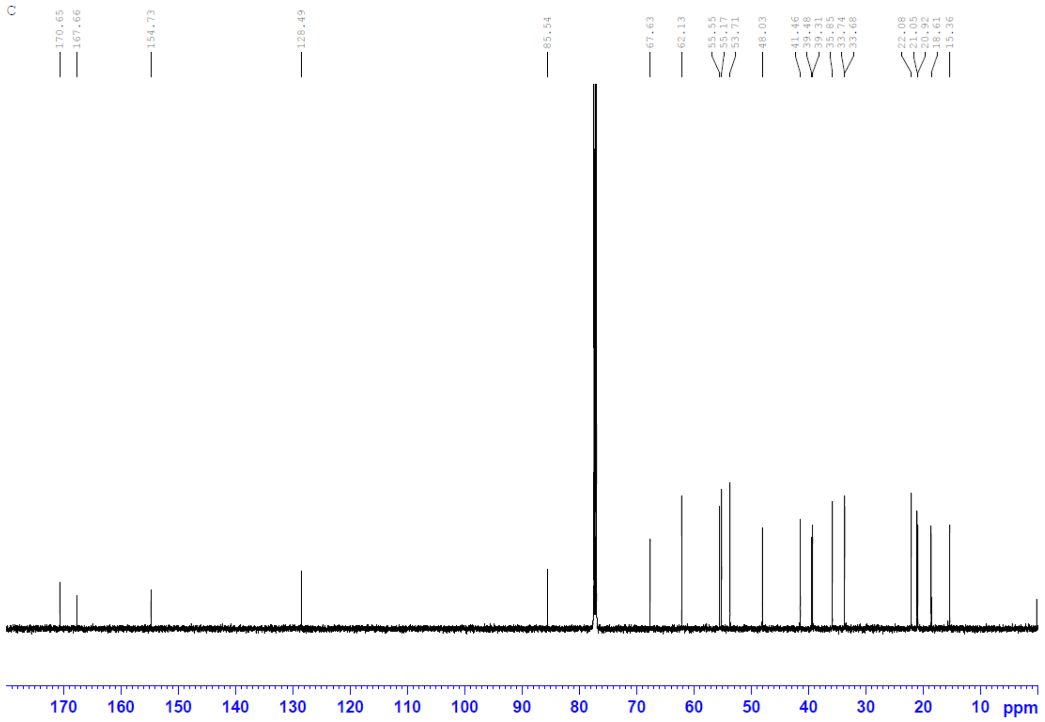

Figure S31. ^{13}C-NMR spectrum of compound **7**.

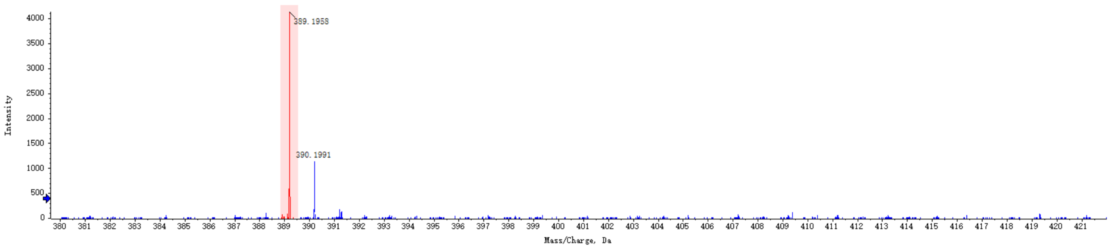

Figure S32. HRESIMS data of compound **7**.

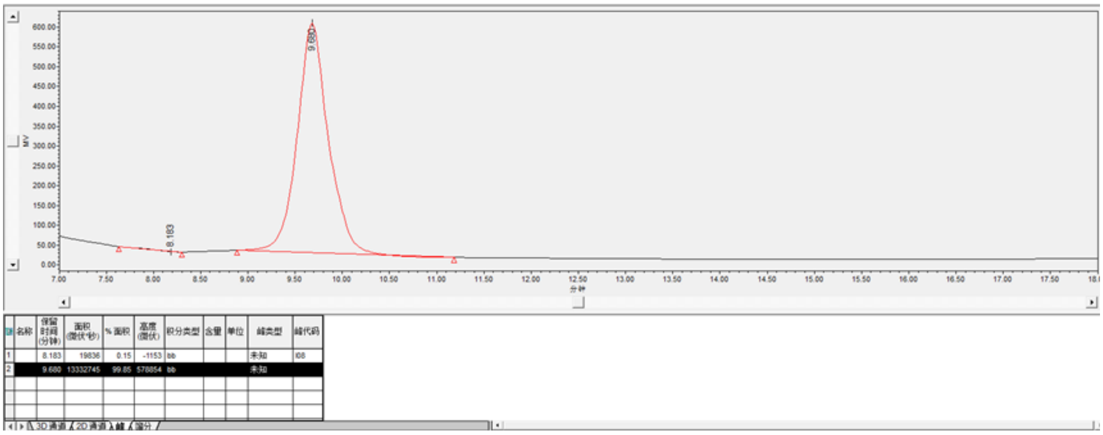

Figure S33. The purity of compound **8**.

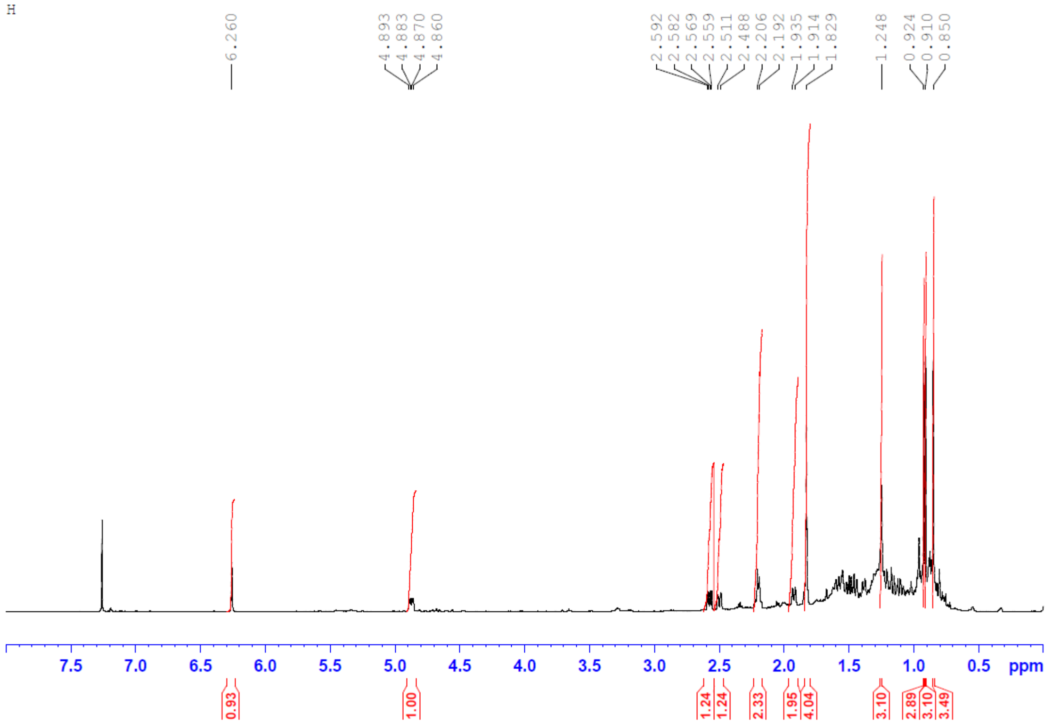

Figure S34. ^{1}H-NMR spectrum of compound **8**.

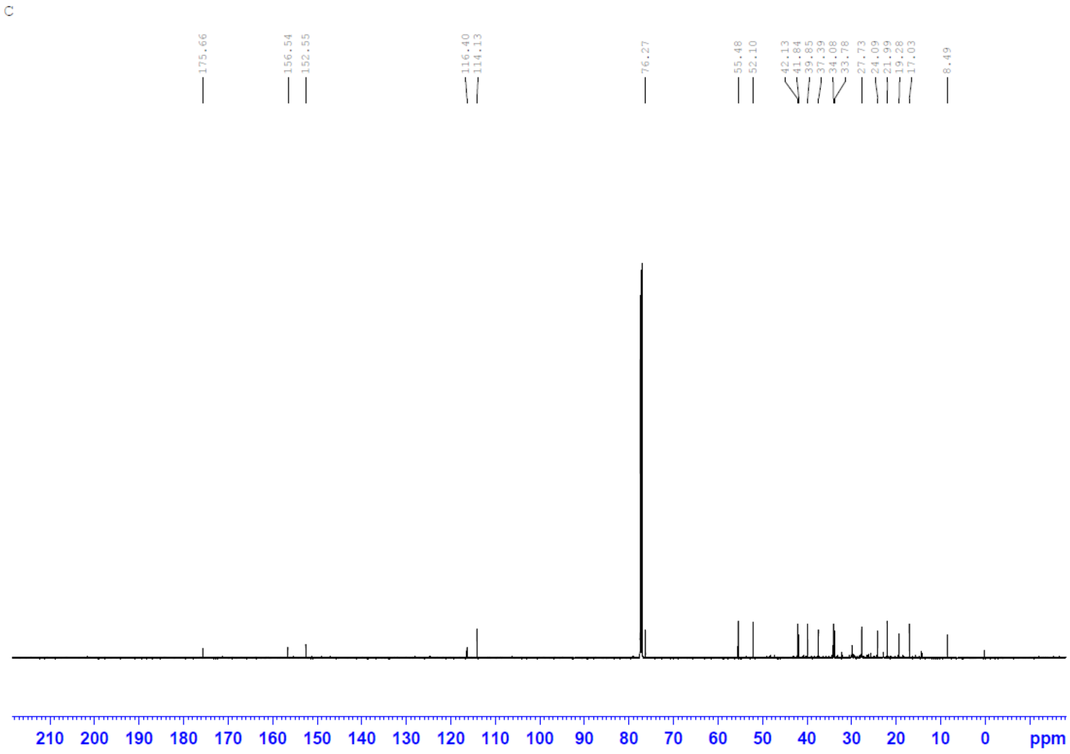

Figure S35. ^{13}C-NMR spectrum of compound **8**.

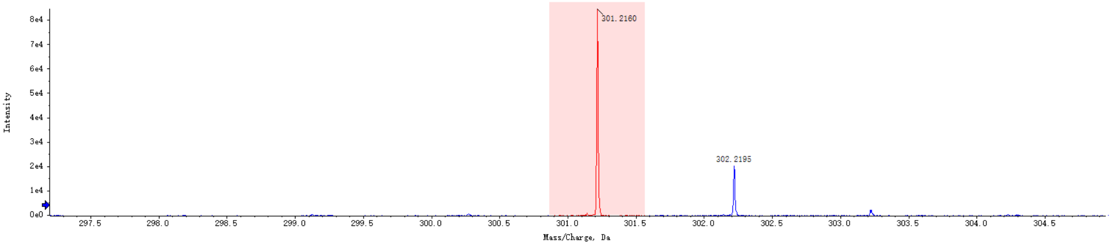

Figure S36. HRESIMS data of compound **8**.

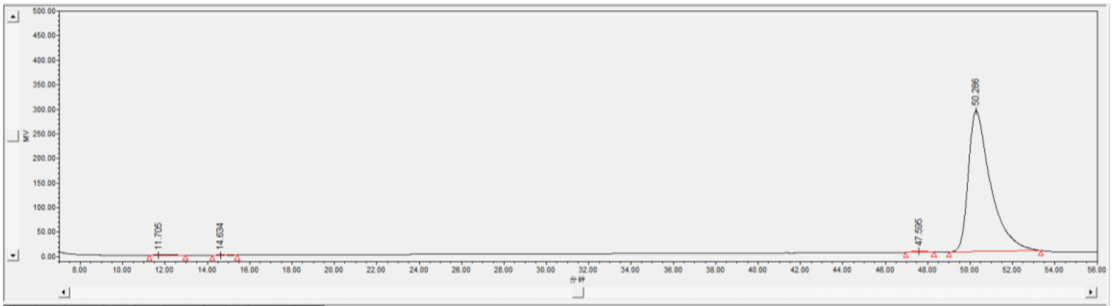

	名称	保留时间(分钟)	面积(微伏*秒)	% 面积	高度(微伏)	积分类型	含量	单位	峰类型	峰代码
1		11.705	45086	0.21	765	bb			未知	
2		14.634	19340	0.09	754	bb			未知	
3		47.595	99225	0.47	2251	bb			未知	
4		50.286	20950910	99.22	287461	bb			未知	

Figure S37. The purity of compound **8**.

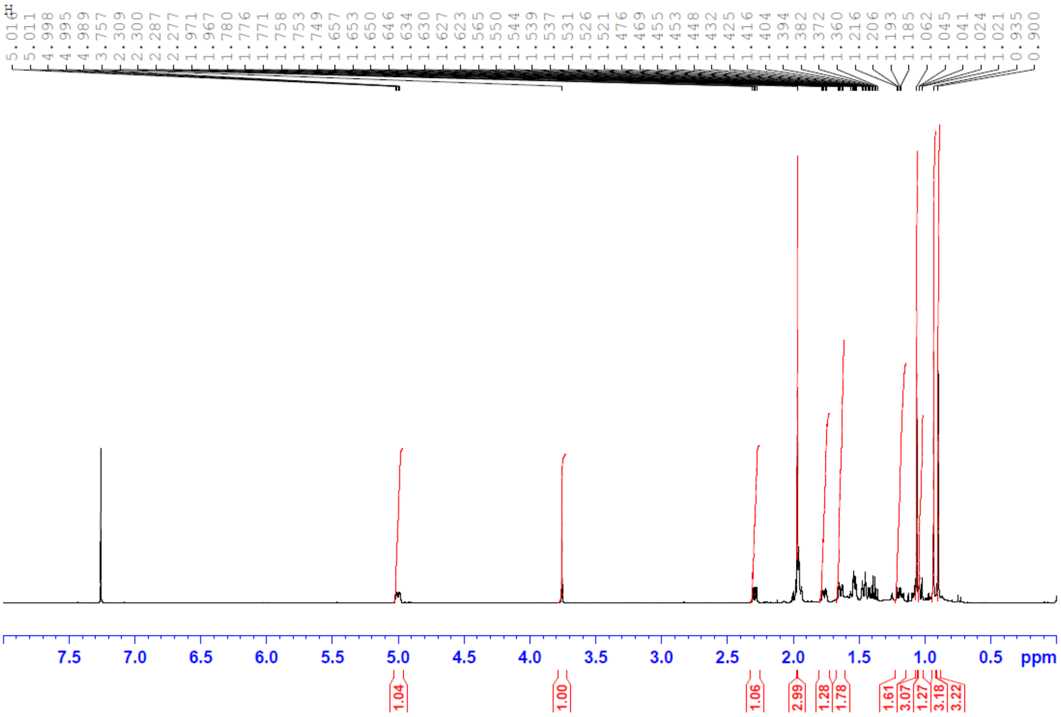

Figure S38. ^{1}H-NMR spectrum of compound **9**.

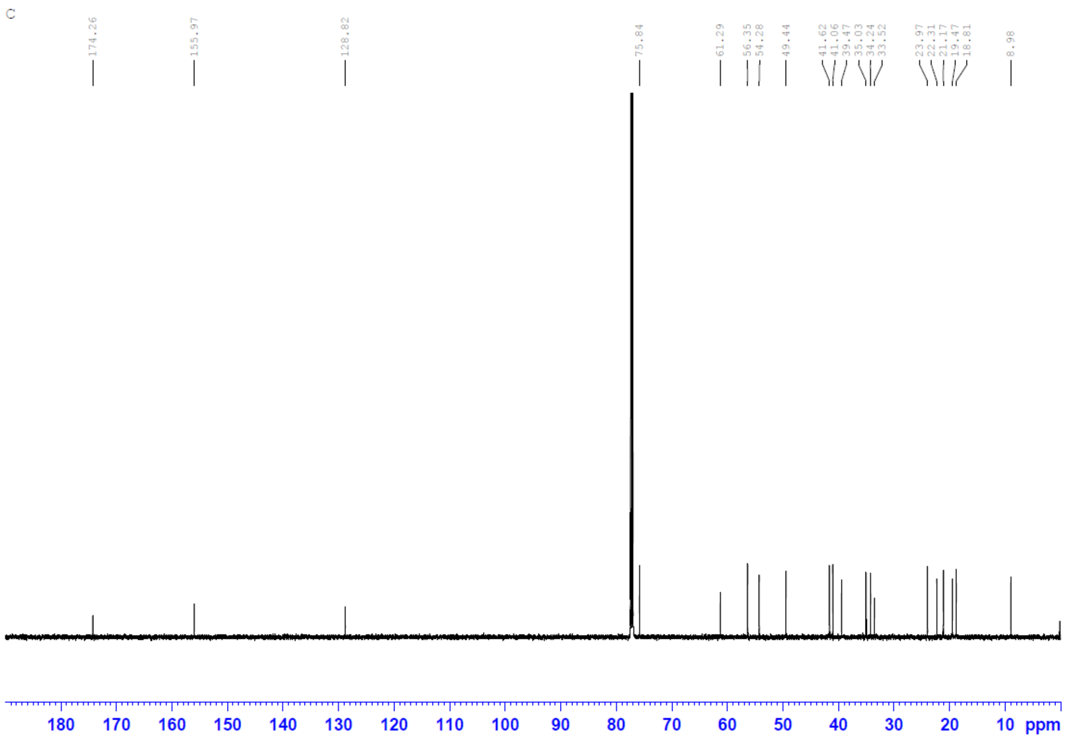

Figure S39. ^{13}C-NMR spectrum of compound **9**.

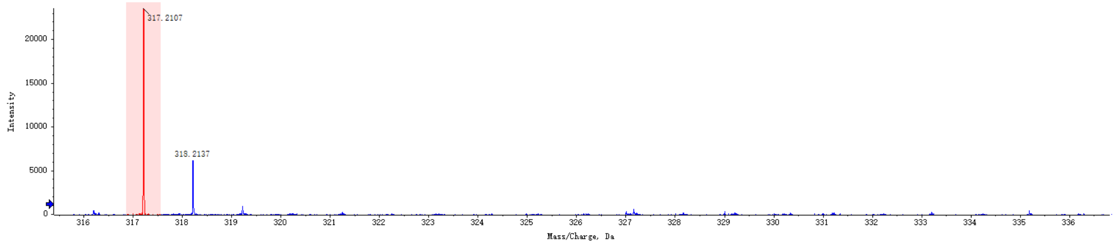

Figure S40. HRESIMS data of compound **9**.

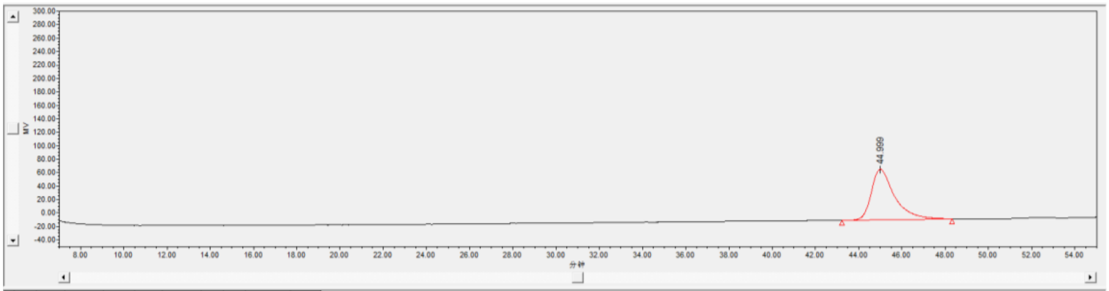

	名称	保留时间(分钟)	面积(微伏*秒)	% 面积	高度(微伏)	积分类型	含量	单位	峰类型	峰代码
1		44.999	5626663	97.49	75453	bb			未知	
2		57.183	144896	2.51	2429	bb			未知	I08

3D 通道 2D 通道 峰 馏分

Figure S41. The purity of compound **9**.

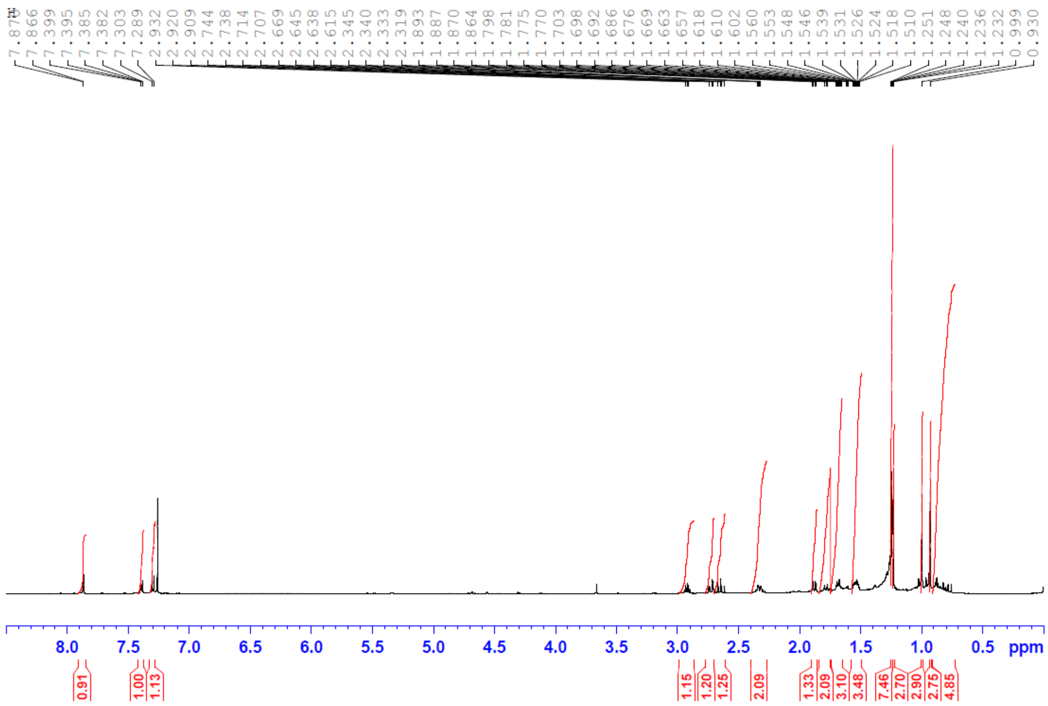

Figure S42. ^{1}H-NMR spectrum of compound **10**.

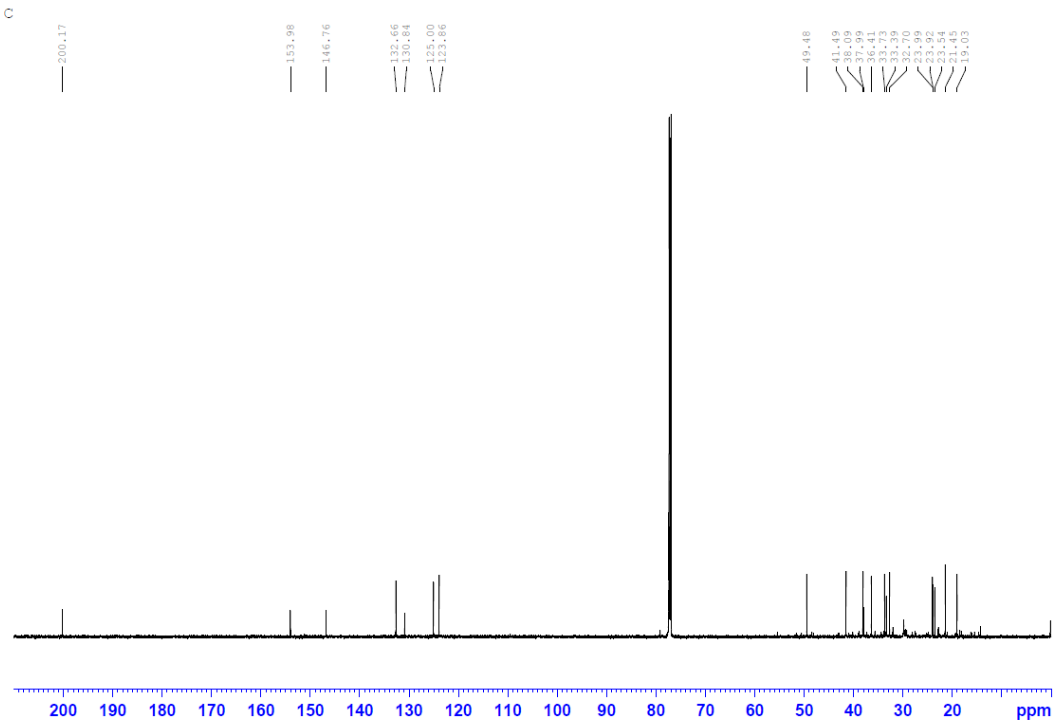

Figure S43. ^{13}C-NMR spectrum of compound **10**.

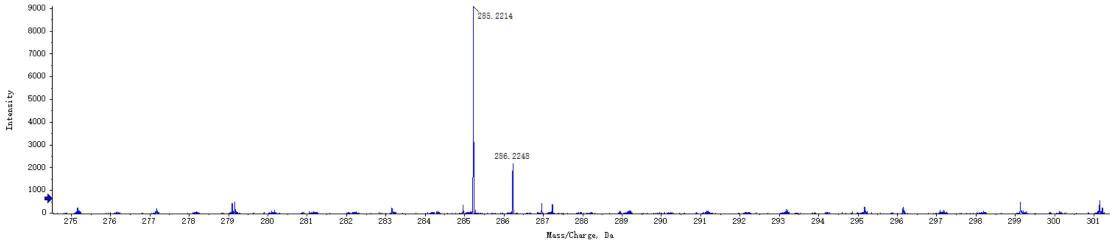

Figure S44. HRESIMS data of compound **10**.

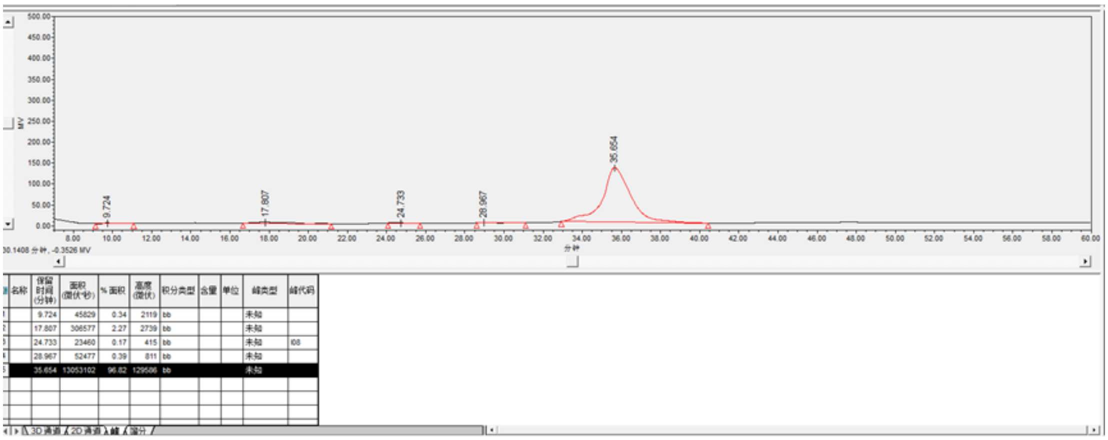

Figure S45. The purity of compound **10**.

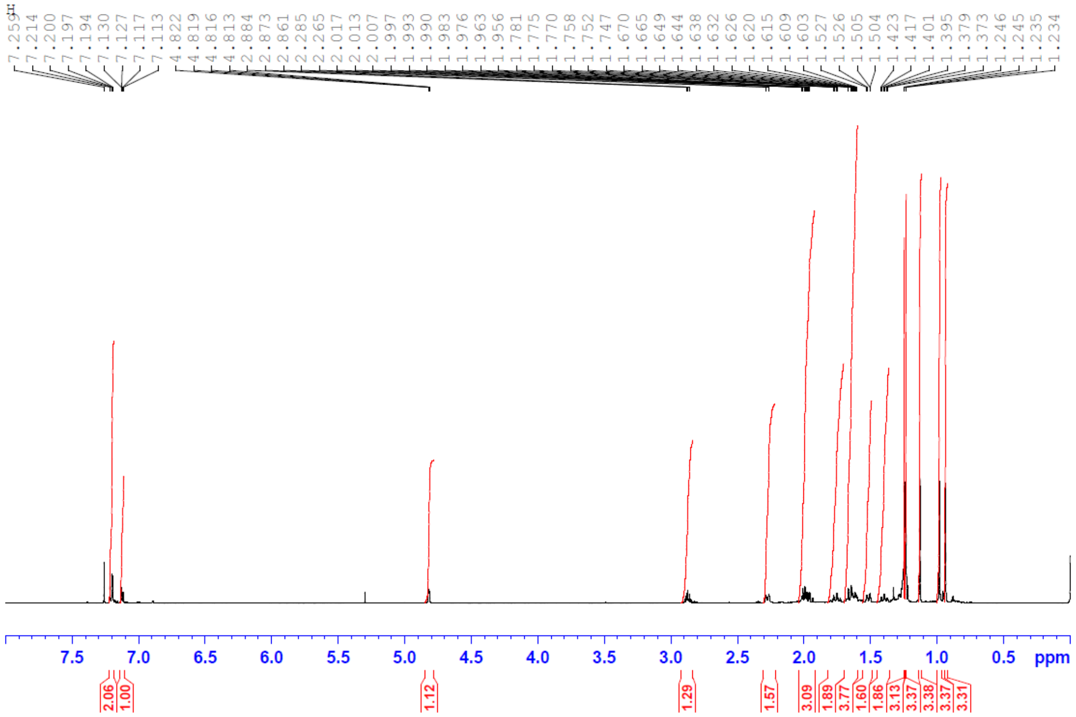

Figure S46. ^{1}H-NMR spectrum of compound **11**.

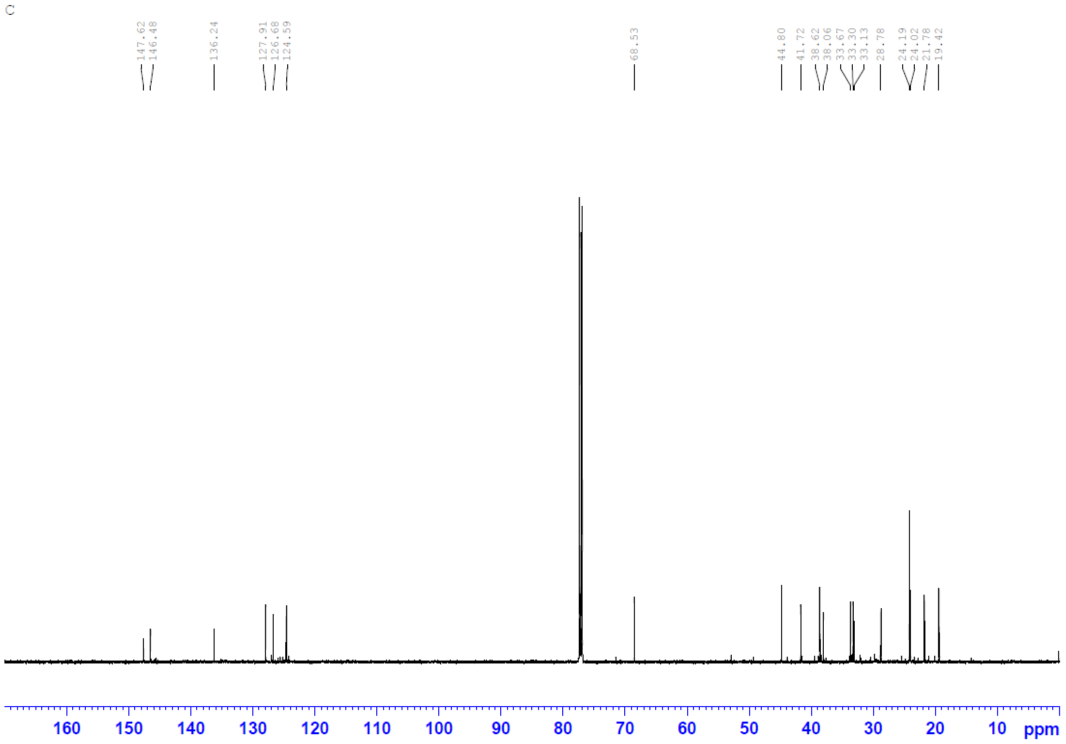

Figure S47. ^{13}C-NMR spectrum of compound **11**.

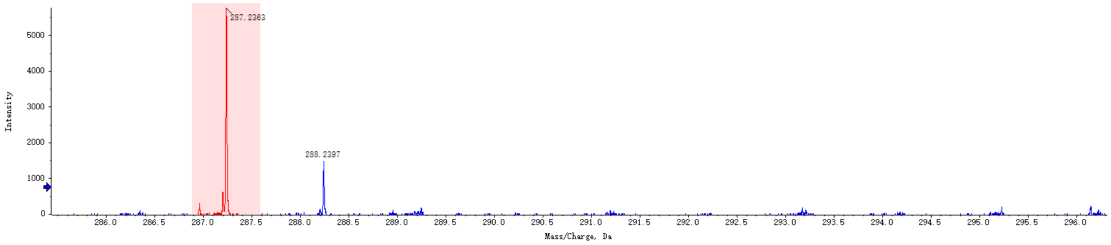

Figure S48. HRESIMS data of compound **11**.

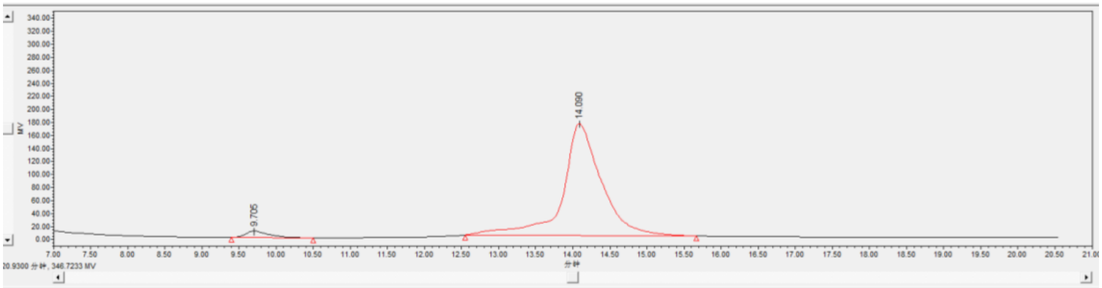

	名称	保留时间(分钟)	面积(微伏*秒)	% 面积	高度(微伏)	积分类型	含量	单位	峰类型	峰代码
1		9.705	197892	3.07	9618	bb			未知	
2		14.090	6250476	96.93	171710	bb			未知	

Figure S49. The purity of compound **11**.
